# Feasibility and acceptability of a life skills and reproductive health empowerment intervention for young newly married women in Rajasthan, India: a pre-post convergent mixed methods pilot study

**DOI:** 10.1186/s40814-025-01720-7

**Published:** 2025-11-15

**Authors:** Lakshmi Gopalakrishnan, Sumeet Patil, Debangana Das, Anshuman Paul, Payal Sharma, Ankur Kachhwaha, Usha Choudhary, Nadia Diamond-Smith

**Affiliations:** 1 University of California, San Francisco, 550 16 Street, 3 Floor, San Francisco, CA 94158 USA; 2 NEERMAN, Unit 3, Mahendra Industrial Coop, Road 29, Sion East, Mumbai, 400022 India; 3 Orange Tree Foundation, No. 08, Imratiya Bera, Paota C-Road, Jodhpur, 342006 India; 4 Vikalp Sansthan, 80, Vinayak Nagar, Ramgiri, Badgaon, Udaipur, Rajasthan 313011 India

**Keywords:** Contraception, Family planning, Empowerment interventions, Gender equality, Health education, Rural and tribal communities, South Asia

## Abstract

**Background:**

In India, newly married young women (18–25 years of age) face high rates of unintended pregnancy. Poor sexual and reproductive health knowledge combined with restrictive social norms leads to adverse pregnancy outcomes among young women, while limited female autonomy prevents them from accessing accurate healthcare information and support. We examined the feasibility and acceptability of TARANG, a life skills and reproductive health empowerment intervention developed for and with young newly married women using a community-engaged approach.

**Methods:**

We report the findings from a convergent mixed-methods, single-group cluster pilot study in Rajasthan, India. We recruited 42 newly married women as participants in our study. Of these, 41 participants (retention rate = 97.6%) completed both baseline and endline surveys in July 2023 and January 2024, respectively. The intervention comprised 16 weekly sessions. Attendance was tracked electronically. We assessed three primary outcomes: feasibility (proportion completing ≥ 50% sessions), acceptability (proportion satisfied/somewhat satisfied), and usefulness (proportion finding TARANG useful/somewhat useful). Twelve participants were interviewed to understand intervention acceptance and usefulness, and 6 program staff and moderators were interviewed to understand implementation barriers. We analyzed quantitative data using descriptive statistics and qualitative data using thematic analysis.

**Results:**

Thirty-five participants completed at least one session, with 73.8% attending at least half of the sessions (6 did not attend any sessions). Among those who attended at least one session, 97.1% were satisfied/somewhat satisfied, and 100% found the intervention useful/somewhat useful. Qualitative findings revealed participants’ appreciation for open discussions on sensitive topics like family planning. The intervention filled knowledge gaps in family planning methods, fostered social connections through rapport building, enhanced sense of agency, and improved relationships with mothers-in-law and husbands. Implementation barriers included women’s workload, cultural norms of extended stays at natal homes, and the need for intense rapport building. These insights informed intervention refinements, including session modifications, increased engagement strategies, and integration of educational videos.

**Conclusion:**

Our pilot study demonstrated high acceptability and feasibility, with multiple benefits for young, newly married women. Our findings informed adaptations to enhance TARANG’s delivery and satisfaction. The effectiveness of TARANG will be tested in a larger cluster randomized controlled trial currently underway.

**Trial registration:**

The study is registered at ClinicalTrials.gov (NCT06320964). Registered retrospectively on 13 March 2024, https://clinicaltrials.gov/study/NCT06320964

**Supplementary Information:**

The online version contains supplementary material available at 10.1186/s40814-025-01720-7.

## Key messages regarding feasibility

- In Rajasthan, India, young newly married women have poor knowledge of reproductive health, face barriers in accessing and using family planning methods, and experience cultural pressures for early childbearing but there is limited evidence on interventions that help them delay childbearing and increase uptake of family planning. A life skills education-based reproductive health empowerment intervention was developed and delivered to young, newly married women in a group format.
- TARANG intervention, developed using a community-engaged approach, was feasible and acceptable among this population.
- Similar interventions could be adapted and tested in similar contexts to address reproductive health needs and empower young, newly married women. Implementation requires addressing barriers including women's workload, cultural norms of extended stays at natal homes, and building strong community rapport to optimize participation and effectiveness.

## Introduction

Marriage is near-universal in South Asia, including India, with a quarter of women aged 20–24 married before the legal age of 18 years [1]. Marriages are “arranged” in this setting, where parents decide on both the timing of marriage and the choice of spouse with minimal input from young women themselves [2, 3]. Patrilocality, a common practice in India, further compounds challenges for newlywed brides, who relocate to their husband’s household to reside with in-laws and experience lower empowerment in terms of their decision-making power, freedom of movement, and autonomy [4, 5]. These restrictions imposed on young married women not only limit their ability to act on health knowledge but also hinder opportunities to discuss fertility desires and family planning methods [6, 7].

In India, societal pressures to prove fertility early contribute to a high prevalence of childbearing immediately after marriage, with 24% of women under 25 giving birth by age 20 [1, 8]. According to India's National Family Health Survey, nearly 66% of young married women (15–24 years) in rural areas do not use any contraceptive method, with traditional methods accounting for 17% of contraceptive use [1]. Prior research suggests barriers that prevent uptake of contraceptives among married women, including poor knowledge about contraception, pro-natal social norms, fear of being labeled “infertile”, son preference, limited spousal communication, fear of side effects, and low women’s autonomy [9–13]. A study found only 30% knew about contraception and 24% understood oral contraception, significantly impacting women's ability to make informed choices about their reproductive health, plan their families, and exercise control over pregnancy timing—essential capabilities for their health and well-being [14].

Further, norms against the use of family planning and pro-natal norms are so powerful that they prevent local community health workers from counseling newly married women about family planning before their first pregnancy [11]. Such deeply rooted norms around proving fertility immediately after marriage often lead to young married women having children earlier than they would have wanted, with negative effects for both the mother and the child, including increased risks of maternal mortality and morbidity, pregnancy complications, low birth weight, stunting, and reduced educational and economic opportunities for young mothers [15]. In addition, many young married women are isolated in their marital homes with low social and peer support and reduced connections with their natal kin, adversely impacting family planning use [7, 16]. Despite the vulnerabilities faced by newly married young women, interventions in India have primarily targeted unmarried adolescents’ empowerment, gender attitudes, and aspirations [17–19]. Without targeted interventions to address the needs of already married newly married women, who are particularly vulnerable to unintended pregnancy, social isolation, and disempowerment, we lack the necessary empirical evidence to effectively support this neglected demographic.

Community-based interventions involving women’s groups to improve women’s health and empowerment have a longstanding history in India with two Indian flagship programs, including the National Health Mission and National Rural Livelihoods Mission. Participatory learning and action groups led by Accredited Social Health Activists (ASHAs) under the National Health Mission have demonstrated improved maternal and newborn health in rural eastern India [20–23]. Concurrently, the National Rural Livelihoods Mission has fostered the formation of over 9 million women’s self-help groups focusing on collective savings, credit, and livelihood activities to empower women individually and collectively [24]. Few studies have explored the effectiveness of layering health and nutrition interventions onto self-help groups (SHGs)—groups of 10–20 women who meet regularly to save money and support each other— reporting improvements in contraceptive use and maternal and child health outcomes, alongside cost-effectiveness [25–27]. Further, a systematic review on Indian community-based women’s group interventions identified 19 randomized controlled trials providing evidence of improved perinatal practices, neonatal survival, immunization rates, and dietary diversity among women and children, while highlighting the lack of impact on changing gender norms and addressing violence against women [28]. Nonetheless, none of these studies specifically targeted the needs of young, newly married young women, echoing the Lancet Commission’s observation on the scarcity of published literature on effective interventions for young adults [29].

Young adults undergo profound physical, cognitive, emotional, and social changes, making them particularly receptive to learning life skills’ into their daily lives. [29] Broadly defined, life skills’ refer to adaptive and positive behaviors that empower individuals to effectively navigate the demands and challenges of everyday life [30]. Life skills’ education trains individuals about risk-taking behaviors and helps them develop communication, assertiveness, self-awareness, interpersonal relationship skills, decision-making, problem-solving, critical thinking, and creative thinking to protect themselves from abuse and exploitation [31, 32]. There is a growing body of evidence that suggests that life skills’ education can play an important role in empowering women to make informed decisions about their sexual reproductive health and change gender attitudes [33, 34] as well as improve mental health outcomes such as anxiety and depression among in-school adolescents [35]. Despite the evidence that life skills’ education has major health benefits, previous attempts at life skills’ interventions from low-income countries have been directed at unmarried adolescents enrolled in school settings, overlooking the critical transition period of marriage [32]. We are aware of one life skills-based couples intervention that is being developed in Pakistan to address fertility norms, mental health stress due to unpaid care work, and decision-making challenges with an objective of improving the quality of life of couples and family units [36]. The life skills’ intervention (PAnKH) implemented in Rajasthan, the site of our study, specifically aimed to empower adolescents, including newly married young women. However, the intervention’s report highlighted formidable obstacles in engaging married women, underscoring the imperative for innovative strategies to reach this underserved demographic [37].

Previous research conducted in India has addressed the family planning and sexual reproductive health needs of married couples, yet neither program employed group-based interventions nor specifically targeted the specific needs of newly married women, as mentioned above. For instance, the CHARM2 program implemented in rural Maharashtra adopted a couple-focused intervention model, emphasizing women’s reproductive autonomy and delivering personalized care and counseling at the couple level, yielding improvements in reproductive and sexual health outcomes [38]. A program dating back over fifteen years, the First-time Parents Project, aimed to enhance the reproductive and sexual health knowledge, autonomy, and social support networks of a diverse group of young married women through integrated interventions during the early stages of marriage and pregnancy. Although positive results were observed in some indicators, findings on reproductive health outcomes were mixed [39]. Except for a handful of interventions [40–42], very few family planning interventions in the Indian context have targeted gender determinants such as deeply ingrained social norms surrounding fertility and gender roles, enhancing spousal communication, male involvement in family planning decisions, promoting women’s autonomy and empowerment over their reproductive health, and addressing concerns about contraceptive side effects.

In summary, the paucity of evidence on this neglected demographic and the persistent challenges faced by young, newly married women, including poor knowledge of sexual and reproductive health, early marriage and childbearing, restrictive gender norms, disempowerment, and poor family planning uptake highlight the pressing need to design and evaluate innovative interventions tailored to their specific context and requirements. Therefore, our team aimed to develop a life skills and reproductive health empowerment intervention using a participatory approach with newly married young women (18–25 years), rooted in their local and cultural context. Additionally, by involving key marital household stakeholders of newly married women, such as husbands and mothers-in-law, we sought to develop an intervention that targets the structural and gendered determinants of sexual reproductive health, including women’s agency/autonomy, couples’ communication on family planning, and inequitable gender norms. Using a community-engaged approach, we developed an intervention, TARANG (Transforming Actions for Reaching and Nurturing Gender Equity and Empowerment) that integrates life skills into reproductive health empowerment, going beyond sexual and reproductive health knowledge to support participants in making informed decisions about their reproductive health and family planning goals.

### Study objectives

This feasibility study aimed to determine whether the TARANG intervention, a life skills’ and reproductive health empowerment program, was ready for testing in a fully powered trial. The objectives were to (1) describe the development of the intervention in detail; (2) evaluate the acceptability, feasibility, and implementation challenges using comprehensive convergent mixed methods; (3) use the findings to inform intervention design and operational details for a larger cluster-randomized controlled trial; and (4) explore implementation barriers and facilitators.

### TARANG intervention

The TARANG intervention integrated life skills into reproductive health empowerment across three areas—empowerment, norms, and sexual reproductive health and wellbeing. These areas were prioritized with our implementing partner based on their extensive field experience with newly married women in Rajasthan. More details published elsewhere [43].

## Materials and methods

### Development of the TARANG intervention for and with newly married women

The curriculum for the intervention was outlined through consultations with our non-governmental organization (referred to as NGO henceforth) implementing partner, Vikalp Sansthan, drawing on their extensive field experience with newly married women in Rajasthan. Subsequently, the curriculum was designed by another organization, Orange Tree Foundation, using a community-engaged approach and topics interconnected to reinforce key concepts. Given the neo-literate target population in rural Southern Rajasthan, sessions were designed to be interactive and activity-driven, delivered in vernacular Hindi with locally relevant content. A comprehensive toolkit including audiovisual aids, illustrated materials, and facilitator guides supported implementation.

We conducted an intervention developmental phase with 38 newly married women in Udaipur district (February–May 2023), evaluating four key parameters: session duration required to deliver the session, participant response, toolkit usability, and participant engagement. Feedback informed refinements to enhance the curriculum’s relevance and linguistic suitability for the target audience before proceeding to the feasibility study reported here (July 2023–January 2024).

### Overview of the TARANG intervention delivery

Two female moderators trained initially over seven days (May–June 2023), with continued supervision and refresher training through October 2023. These moderators facilitated four village-based groups, with ongoing NGO supervision. The intervention comprised an introductory rapport-building session followed by 16 group sessions over six months, using practical activities to build understanding of reproductive health, gender norms, and rights (see Additional file 1). The intervention included light-touch complementary sessions for husbands and mothers-in-law to foster household support (reported elsewhere) [44].

### Study design and setting

This convergent mixed methods study consisted of a one-group design with pre- and post-intervention data collection to examine the feasibility and acceptability of the intervention. The study was conducted in four villages^1^ of Kumbhalgarh block, Rajsamand district, Southern Rajasthan (July 2023–January 2024). This district has low modern contraceptive use, with female sterilization being the predominant method, indicating limited adoption of reversible contraceptive methods [45].

This pilot study enrolled 42 newly married women. Eligibility criteria were: (1) aged 18–25 years, (2) married within the past year, (3) living with husband and mother-in-law for at least six months, (4) residing in the study villages, and (5) willing to provide verbal consent. All participants provided audio-recorded verbal informed consent.

### Data collection

**Fig. 1:**
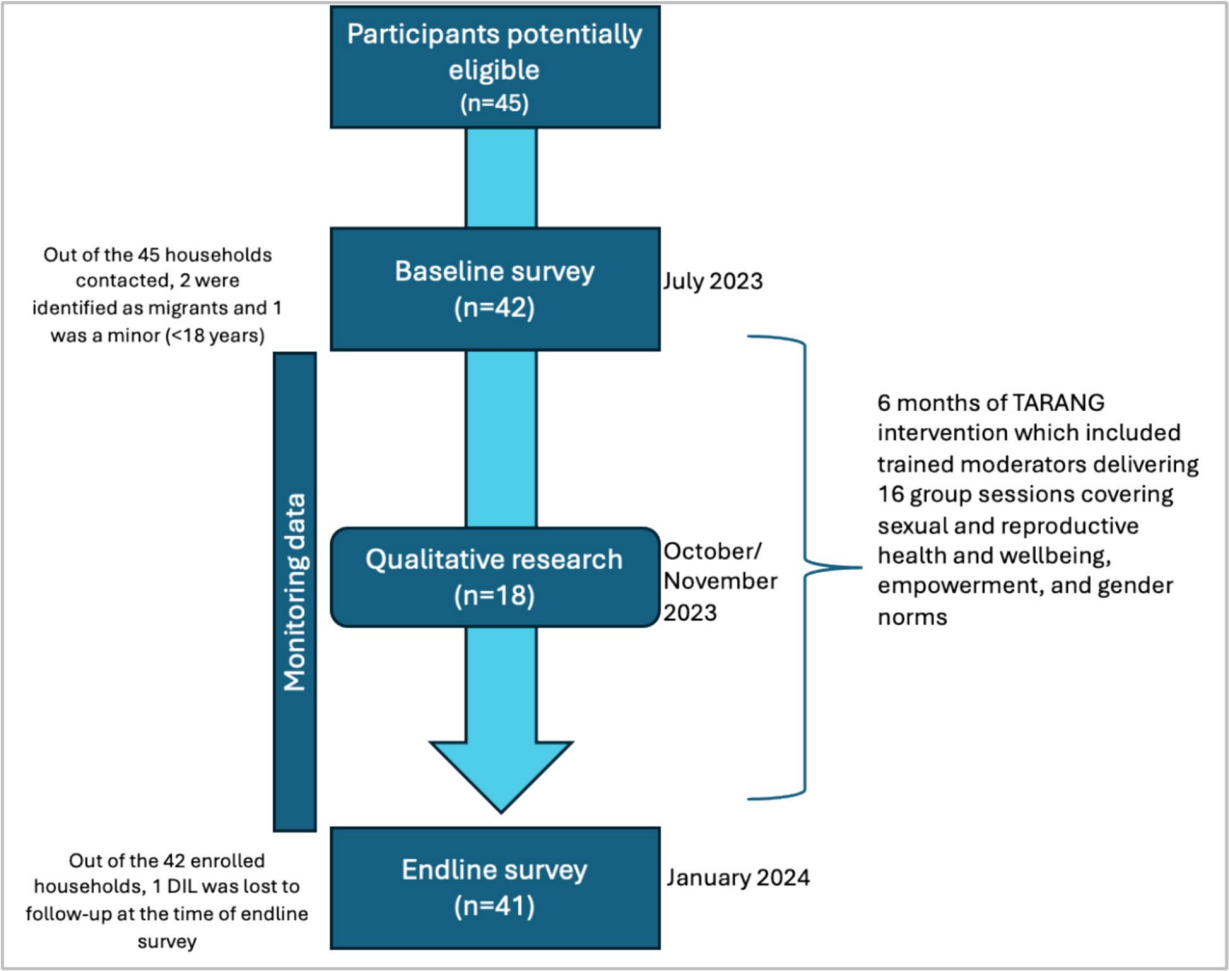
Timeline of TARANG intervention sessions and different modes of data collection

The intervention timing and data collection are shown in Fig. 1.

### Quantitative surveys

Baseline data were collected before participants’ first session in July 2023, and participants evaluated the program after the final session (January 2024). These data were collected using structured computer-assisted personal interviews conducted by sex-matched trained enumerators (~ 60–70 min). Surveys included domains such as fertility preferences and decision-making, use of family planning methods, pregnancy and birth history, women’s empowerment, relationship quality; see Additional file 2 for full details.

### Qualitative data

Using maximum variation sampling, we selected 12 participants for in-depth interviews based on intervention attendance (including both < 25% and > 50% attendance) to capture diverse experiences. We also interviewed 6 NGO staff involved in implementation. In October 2023 (mid-intervention between July 2023 and January 2024), the first author (LG) and UC conducted interviews in Hindi using a semi-structured guide (Additional file 3) in private settings at participants' homes. Interviews averaged 35 min, were audio-recorded with verbal consent, and transcribed.

### Monitoring data to track attendance of participants

An electronic monitoring software was developed and deployed as a mobile application for moderators. Participants’ session attendance and reasons for absence were monitored by moderators through an electronic application (referred to as “monitoring data”).

### Quantitative measures

Our primary outcome measures included

1. Feasibility (proportion of participants who attended at least 50% intervention sessions)
2. Acceptability (proportion of participants completely satisfied/somewhat satisfied with TARANG intervention)
3. Usefulness (proportion of participants who found TARANG intervention useful/somewhat useful)

Retention rates were defined as women enrolled at baseline through the end of the study and are presented as percentages. Quantitative data were summarized as proportions using Stata 15.1. [46].

Sample size justification: the sample size for this feasibility study was determined based on our primary objective of estimating the feasibility of intervention delivery, specifically the proportion of participants attending at least 50% of sessions, with adequate precision to inform a future definitive trial. Without established attendance benchmarks from similar interventions in this population, we conservatively assumed an attendance rate of 60–70% to calculate our sample size. Following the confidence interval approach for pilot studies [47], with 40 participants, this would yield a 95% confidence interval of approximately ± 15% (45%–75% if attendance was 60%), providing sufficient precision to determine feasibility thresholds for a future trial [48]. For the qualitative component, this sample size is informed by established guidelines for qualitative research, where saturation typically occurs within 12 interviews, with basic thematic elements emerging as early as 6 interviews [49].

### Qualitative themes and analysis

We analyzed in-depth interview transcripts line-by-line using a codebook. We developed a coding framework deductively based on the interview guide, iteratively refined it with the inductive codes following the coding of initial transcripts. A team of three researchers (DD, AP, and LG) initially double-coded at least 10% of the transcripts using the codebook in Dedoose version 9.0.107 [50]. The first author (LG) queried and analyzed the code reports and developed themes along with illustrative quotes using thematic analysis [51]. This was followed by review and editing by co-authors (DD, AP, UC). This included members of the implementation and research team in India, ensuring the interpretations accurately reflected the nuances and complexities of Rajasthani culture.

### Mixed methods integration

We employed a convergent mixed-methods approach, with the interview findings to complement and elucidate the quantitative results [52]. We present the quantitative and qualitative findings separately and integrate them using a joint display in the results section, followed by a synthesis of both in the discussion to derive insights [53, 54].

## Results

### Participant characteristics at baseline

Across the four villages, we identified all eligible young newly married women, totaling 45 individuals. The high recruitment rate was potentially because the intervention was culturally relevant and community moderators’ preliminary outreach activities prior to formal recruitment. Of these, three did not meet study eligibility criteria (see Fig. 1). All 42 eligible women were invited to participate in the study, and all agreed. Ages ranged from 18 to 24 years (mean 20.3, SD ± 1.6); most (78.5%) had a marriage that was arranged by their parents. About 38% of participants had received some primary schooling, over a third had secondary education, and about 14% had received no education at all. Most participants in our sample belonged to the indigenous communities identified as Scheduled Tribe (61.9%) or historically marginalized groups identified as Scheduled Caste (14.2%).^2^ The majority (92.9%) wanted two or more children, and about 71.4% wanted to wait at least two years before having their first child. About a quarter (~ 23.8%) reported ever having used a method of contraception. Sociodemographic and fertility preference-related characteristics are summarized in Table 1.

**Table 1:** Baseline demographic characteristics and fertility preferences of intervention participants (*N* = 42), interview participants (*N* = 12), and program staff (*N* = 6)

	Surveys *n *(%)	Participant interviews *n* (%)	Program staff and moderators’ interviews *n *(%)
Total participants	*N* = 42	*N* = 12	*N* = 6
Male	–		
Female	42 (100%)		
Age in years, mean (SD)	20.3 (1.6)	21.3 (2.4)	44.8 (7.5)
Marriage type			
Arranged	33 (78.6)	–	–
Love	9 (21.4)	–	–
Education			
No formal education	6 (14.3)	–	
Primary education (1–8)	16 (38.1)	5 (41.0)	
Secondary education (9–12)	14 (33.3)	4 (33.0)	
College (Bachelor)	6 (14.3)	3 (25.0)	6 (100)
Employment status			
Never employed	25 (59.5)	3 (25.0)	
Not currently employed	15 (35.7)	–	
Currently employed	2 (4.8)	9 (75.0)	6 (100)
Caste			
Scheduled caste (SC)	6 (14.2)	6 (50.0)	–
Scheduled tribe (ST)	26 (61.9)	4 (33.3)	–
Other backward class (OBC)	5 (11.9)	2 (16.7)	–
General category	3 (7.2)	–	–
Missing	2 (4.8)	–	
Preferred number of children			
No preference	3 (7.1)	–	–
Two or more	39 (92.9)	–	–
Preferred time to start family			
No preference	5 (11.9)		
Less than 1 year	7 (16.7)		
Two or more years	30 (71.4)		
Ever used contraception	10 (23.8)		

### Quantitative results on feasibility and acceptability

Attendance to the groups was high: 73.8% (31 out of 42 participants) attended more than 50% of the sessions (see Table 2). Twenty-six percent (11 out of 42 participants) attended fewer than 50% of the sessions. One person who was lost to follow-up attended one session. Of those who attended at least one session, the median number of sessions attended was 14 out of 16, i.e., 50% of the women attended at least 14 or more sessions (Interquartile range: 11–16), suggesting strong feasibility. Six of the 42 enrolled participants (14%) did not attend any session. The reason for non-attendance over the 6-month period included: temporary migration for work outside the villages. It is likely these six participants left immediately after the baseline survey and only returned around the time of the endline survey.

**Table 2:** Primary outcomes of TARANG intervention

Primary outcomes	*N* (total sample size)	Indicators	*n* (%)
Feasibility^a^	42	Proportion of participants who attended at least 50% of the sessions	31 (73.8%)
Acceptability	35^b^	Proportion completely satisfied/somewhat satisfied with TARANG intervention	34 (97.1%)
Usefulness	35^b^	Proportion who found TARANG intervention useful/somewhat useful	35 (100.0%)

^a^Calculated using the monitoring data/mobile application filled by moderators and NOT the endline survey

^b^Calculated based on 35 participants who attended at least one session and those who completed the endline survey (please note one person was lost to follow-up in endline survey)

In terms of acceptability, among those who attended at least one session and were surveyed at endline, 97% reported being satisfied/somewhat satisfied with the intervention. This high satisfaction rate among actual participants indicates reasonably high acceptability of the program content and delivery among those who engaged with it. In terms of usefulness, among those who attended at least one session and were surveyed at endline, all participants who attended at least one session found the TARANG intervention either to be useful or somewhat useful.

Participant satisfaction with TARANG was high (see Table 3). Of 42 participants recruited at baseline, 41 were retained at the end of the study (97.6%), as defined by completion of follow-up surveys. One participant was lost to follow-up due to migration after attending 1 session. Among those who attended at least one session and completed endline surveys (*n* = 35), satisfaction indicators were consistently high, with most participants reporting improved decision-making confidence (85.7%) and enhanced spousal communication about family planning (97.1%).

**Table 3:** Other outcomes assessed for the pilot intervention

Other outcomes assessed (%)	*N*	*n* (%)
Retention rate^a^ (%)	42	41 (97.6%)
Very likely recommend a friend to join the TARANG intervention	35	35 (100.0%)
High level of connection with the other participants during the TARANG intervention sessions	35	35 (100.0%)
Perceived the session was just right	35	35 (100.0%)
Perceived interval between two sessions in the TARANG intervention was just right	35	34 (97.1%)
Perceived right number of sessions held in the TARANG intervention	35	34 (97.1%)
Perceived information from TARANG intervention could be applied in their daily life to great extent/some extent	35	35 (100.0%)
Perceived confidence about making decisions about timing of children and health after participating	35	30 (85.7%)
Perceived positive changes in themselves regarding making decisions about having children and family planning after participating in the TARANG intervention	35	35 (100%)
Perceived positive impact of TARANG intervention on communication and understanding between participant and their husband regarding family planning	35	34 (97.1%)
Liked the TARANG intervention	35	35 (100%)
Want sessions of TARANG intervention to be held in their village	35	35 (100%)

^a^Of the 42 participants enrolled at baseline, 1 participant was lost-to-follow-up at endline due to migration. Please note all outcomes are assessed using data from 35 participants who were followed up and attended at least one session

### Qualitative results

Table 4 outlines the themes, including intervention acceptability, usefulness, implementation barriers and facilitators, and suggested improvements:

**Table 4:** Overview of themes presented

Themes	Overview
Theme 1: Acceptability of the intervention	• Acceptance of the intervention among participants
Theme 2: Intervention’s Usefulness	• Knowledge • Decision-making and agency • Social networks • Improved relationships
Theme 3: Barriers and facilitators (Participant perspectives)	• Workload, daily routines, household responsibilities • Some resistance from husbands/fathers-in-law • Logistical challenges • Strong desire to attend meetings to navigate barriers
Theme 4: Barriers and facilitators (Moderator perspectives)	• Challenges in recruiting newly married women for the study • Strong desire to navigate barriers and attend meetings • Time constraints as barrier • Initial shyness and need for rapport building • Cultural events and festivals • Newly married women’s extended period of stay at their natal homes
Theme 5: Suggestions for intervention improvement	• Themes in the meetings/sessions • Length/time of the meetings • Conduct of sessions by moderators • Intervention delivery to include videos

### Theme 1: Acceptability of the intervention

Nearly all the participants reported that attending meetings made them feel “good”. The participants elaborated that meetings offered a sense of ease to sit and talk about family planning, menstruation, and other topics.

“Being a part of the program has provided me with new information that I didn't have before. I have been connected with new people, who I did not know earlier. I've gained new knowledge since joining.” – Newly married woman #10.

Some women expressed appreciation for the TARANG intervention, as it equipped them with essential practical knowledge that had previously not been provided to them in any form, as well as increased mobility. They explained that given the gender norms in their villages, they were never allowed to step outside their homes to attend such sessions previously.

“I did not know before. Because we were not allowed to step outside the house. We used to go to school. Right after that, we used to go to the fields for work. They [parents] didn’t allow us to go anywhere.” Newly married woman #10.

Interviews with NGO staff and moderators underscored the evident necessity for an intervention such as TARANG for newly married young women, reflecting the program’s acceptance within the communities.

“There is definitely acceptance within people. The young married couples I talked with have found the need for such information. Young married couples don't have this kind of information. ….If they don't have this information and are able to grasp such information, then it is acceptable.” NGO staff #2.

“People in my village share what they learn in meetings with each other, and it feels like they are learning something from us. Whatever we tell them, they are learning something new… Participants agree to continue with the program. I am receiving support from daughters-in-law, mothers-in-law, and even from husbands and fathers-in-law.” NGO moderator #1.

### Theme 2: Intervention’s usefulness

Participants reported benefits from TARANG in five key areas: knowledge, decision-making, social connections, spousal communication, and family relationships.

#### Usefulness: knowledge

Participants gained understanding of contraception, menstruation, and birth spacing. Most discussed learning about various contraceptive methods and the importance of child spacing.

“We discussed about different contraceptive options—condoms, pills,… There should be a three-year gap between the birth of two children. When you don’t space children, the body gets weak.” Newly married woman #12.

Participants understood the importance of three-year birth spacing for maternal and child health.

“There should be a gap of two to three years between children. It means the first one should grow up, and preparations for the second one should be made, taking care of oneself…It's good that we discuss things related to our body parts; that's correct.” Newly married woman #3.

Participants demonstrated awareness of unprotected sex risks and the importance of contraception*.*

“Yes, I feel worried; there's fear. Sometimes, engaging in relationships without contraceptives, child maybe conceived….” Newly married woman #6

Moderators noted the effectiveness of interactive teaching methods, particularly the “seed activity” which helped participants understand the biological basis of child sex determination.

“When we conducted sessions, they didn't know before how pregnancy occurs, what a boy is like, and what a girl is like. Through activities, they are getting this kind of information. Through activities like “seeds”, they understand that there are two types of seeds, one for men and one for women. They now understand menstruation, are aware of their health, and know the benefit of spacing in children, including family planning awareness”. NGO moderator #1.

#### Usefulness: decision-making process and sense of agency

Few participants reported increased autonomy and agency in reproductive health decision-making, shifting from solely seeking family approval to making independent choices. They developed confidence in both personal and joint decision-making with spouses.

“Earlier, I couldn't make decisions on my own.. And if it concerns me, I can make my own decision.” Newly married woman #5

A few participants became more proactive about family planning, considering factors like birth timing and spacing.

“Now we think differently—When should we have a child, how much age gap should be there between children, etc.” Newly married woman #3

#### Usefulness: new connections and establishment of social networks

Participants valued the social connections formed during sessions, which enabled peer support and information sharing.

“We make friends by attending the meeting. We learn how to talk, get to know each other, and understand our bodies..” Newly married woman #3

#### Usefulness: relationship with husbands/couples’ communication

A few participants reported sharing session information with husbands, improving couple communication about reproductive health and family planning. “We both take each other's opinions. We work by consulting each other. It was a bit less at first, but now it's fine” Newly married woman #3.

#### Usefulness: relationship with mothers-in-law

Participants noted improved relationships with mothers-in-law, possibly due to dedicated intervention sessions that promoted mutual understanding.

“Like, when we are going to the meeting, my mother-in-law and husband listen to me, and I also listen to them. Everything is going well….In the session, we learned that one should consider their daughter-in-law as a daughter. For example, our mother-in-law considers her daughter, who got married, as a daughter-in-law, just like they (mothers-in-law) should consider us daughter-in-law as their daughter, and that's how we receive love.” Newly married woman #3.

### Theme 3: barriers and facilitators (participant perspectives)

#### Workload, daily routines, and household responsibilities

Participants faced several significant barriers to attending sessions, primarily revolving around their demanding daily routines. Most participants juggled extensive household chores with agricultural labor or cattle rearing, leaving little time for rest or additional activities. As one 18-year-old participant described, her day was consumed by making roti, tending to livestock, and farm work:

“I make roti [bread], fetch fodder for the buffalo, and eat roti [bread]. Bringing fodder for the goat, then taking a two-minute rest, going to the farm at three-four o’clock, and in the evening, making roti again” Newly married woman #9.

Family care obligations created additional challenges for some participants. These included caring for sick relatives or assisting family members with various tasks, often requiring them to manage responsibilities across multiple households. One participant explained her situation: "*I have to take care of both the houses…I go to my father's place at night and return in the morning.*" *Newly married woman* #6.

#### Some resistance from husbands/fathers-in-law

While most families were supportive, some participants encountered resistance from husbands or fathers-in-law. These family members sometimes discouraged attendance, preferring women to focus on household duties. As one participant noted,

"My husband says… I should do household chores rather than sit in the meetings." Newly married woman #1.

NGO moderators confirmed this challenge, noting that some fathers-in-law objected to women’s participation. Their objections were based on fathers-in-law’s own health concerns, such as pre-existing cardiovascular conditions that required care and attention from the women in their household.

#### Logistical challenges

Logistical challenges also impacted attendance, with some participants living far from the meeting venue. For some groups, members needed to travel up to 15 min to reach the location.

#### Strong desire to navigate barriers and attend meetings

Despite these various obstacles, many participants demonstrated strong commitment to attending the sessions. They actively managed their time around their schedules, sometimes attending without explicit permission, and viewed the meetings as valuable enough to overcome these challenges. As one 21-year-old participant explained: “Work is never-ending… We allocate two hours for the meeting.” This determination highlighted the perceived value of these sessions for the participants.

### Theme 4: barriers and facilitators (NGO staff and moderator perspectives)

#### Challenges in recruiting newly married women for the study

A significant cultural practice in Rajasthan known as “gauna” or “aana” affected recruitment. This custom involves newly married brides transitioning to their in-laws’ home after a variable period, ranging from months to over a year post-marriage, rather than immediately after wedding ceremonies. This practice made it particularly challenging to identify eligible households that met all study criteria—married women aged 18–25 who had lived with their husband and mother-in-law for at least six months, with marriages within the past year. Despite these challenges, the team managed to recruit sufficient households for the pilot study.

#### Time constraints as barrier

Women's demanding daily routines, including household chores and labor-intensive agricultural work, often made it difficult for them to attend sessions regularly. Moderators noted that many women prioritized household responsibilities over meetings, fearing domestic work would suffer. The seasonal grass-cutting period in Rajasthan, primarily undertaken by women, added another layer of time constraints during the qualitative study period. “But it's quite challenging to call women for meetings as they are busy with fieldwork. Sometimes when I invite them to a meeting, they respond, we must go home to our guests. The biggest challenge is that 90 per cent give more priority to household chores, fearing that if they attend the meeting, the household work will suffer, due to a lack of time.” NGO moderator #1.

#### Initial shyness and need for rapport building

Moderators observed that participants were hesitant to speak up during group sessions due to feelings of embarrassment. To address this, moderators worked with mothers-in-law to encourage women's participation and help overcome this initial reluctance. As one moderator noted: "Initially, newlywed brides wouldn't talk to us, and wouldn't even disclose their names… Due to shyness, they would hide in someone else's house." NGO moderator #2.

#### Cultural events and festivals, weddings impacted attendance

Certain months, particularly during Sawan (monsoon), and festivals like Holi and Diwali, saw increased participation in religious and cultural activities, making it difficult to maintain consistent session attendance. Furthermore, newly married women’s extended stays at their natal homes, a common practice during the transition period, affected their ability to attend sessions regularly. To facilitate attendance, moderators often personally escorted participants to sessions, sometimes even providing transportation via scooter when necessary.

#### Newly married women’s extended period of stay at their natal homes

The transitional period for newly married brides, as they acclimatized to their marital homes, often resulted in extended stays at their natal/parental homes, presenting a significant barrier to their consistent attendance at sessions. A staff member explained: “Daughter-in-law visits their parents quite frequently, due to being newly married; she stays in the in-laws' house for some time and sometimes in her parental home.” NGO program staff #2.

To overcome this barrier, moderators often took extra steps, including personally escorting participants and even providing transportation via scooter when necessary. As one moderator noted: “Our colleagues bring them sitting on a scooter, many times when the husbands do not have the resources, both the husband and wife are brought for the session.” NGO moderator 3.

### Theme 5: suggestions for intervention improvement

A few intervention improvement themes emerged from interviews with both participants and NGO staff.

#### Themes in the meetings/sessions

Most participants agreed that the intervention effectively addressed their learning desires and covered relevant topics. NGO staff emphasized the importance of sensitive content delivery, as one staff member noted: “The content is right. It is essential how sensitively it is being taken to them… The content is based on the subject we are talking about." NGO program staff #2.

#### Length/time of the meetings

Regarding session duration, several participants expressed interest in longer meetings to allow for more detailed discussions. As one participant suggested: “It should be increased. The more time we sit for the meeting, the more we will talk.” Newly married woman #3.

NGO moderators supported this view, noting that extended sessions would better accommodate participants’ sharing personal experiences.

#### Conduct of sessions by moderators

The moderators’ delivery style received widespread appreciation. Participants commended their ability to explain topics in accessible language and contextualize information effectively. As one participant noted:

“Madam's way of explaining is correct; she explains to us in our language. She keeps the sessions very lively—fun and not serious. I like her teaching very much.” Newly married woman #5

#### Intervention delivery to include videos

Regarding intervention delivery methods, participants strongly supported incorporating more videos, viewing them as engaging learning tools similar to stories. One participant noted: "Watching videos will help in understanding, it's like a story only.” Newly married woman #5.

Participants appreciated diverse instructional approaches, including pictures, stories, case studies, and audio. NGO staff agreed that videos enhanced engagement and understanding, with one moderator suggesting: “After showing them the videos, we can discuss them with them. This way, their interest will be maintained in watching the videos, whose language is easy, and understandable.”

#### Data integration

Table 5 lists the primary outcomes, with exemplar quotes from interviewees and corresponding quantitative statistics.

**Table 5:** Joint display table for integration of quantitative and qualitative findings

Implementation outcomes	Qualitative results and exemplar quotes from participants	NGO/program staff interview results	Quantitative results from monitoring data	Quantitative results from those who completed endline surveys and attended at least one session	Mixed methods interpretation
Feasibility	While participants expressed enthusiasm for attending meetings, they faced several challenges including demanding daily routines and distance to meeting venues. Although some encountered resistance from husbands or fathers-in-law, their commitment to participation remained strong. As one participant explained: “No, still, we manage time for the meeting. There is no big deal with work, as Work is never-ending… We allocate two hours for the meeting. If there is any work, we complete it before attending.” This determination highlighted participants' ability to navigate barriers and prioritize session attendance despite obstacles	NGO staff and moderators confirmed the program's feasibility while acknowledging several implementation challenges. These included festival schedules, women's limited availability due to household duties, extended stays at natal homes, and initial shyness requiring rapport building. Despite these barriers, they noted encouraging support from families, as one NGO staff member shared: "*Participants agree to continue with the program. I am receiving support from DIL, MIL, and even from husband and father-in-law.*"	• 73.8% (31 of 42 participants) attended more than 50% of the sessions	• 100% (35 of 35) participants liked the TARANG intervention • 100% (35 of 35) participants wanted sessions of TARANG intervention to be held in their village	Despite the noted barriers, we observed an overwhelmingly strong alignment between quantitative findings and qualitative reports from participants and staff
Acceptability	Participants overwhelmingly appreciated the TARANG intervention, particularly valuing the opportunity to discuss previously taboo topics like family planning and menstruation. They also actively shared their newly acquired knowledge with others in their community. As one participant expressed: “Being a part of the program has provided me with new information that I didn't have before. I have been connected with new people, who I did not know earlier. I've gained new knowledge since joining.”	NGO staff and moderators emphasized TARANG's importance for newly married young women, noting strong community acceptance and the program's role in addressing critical knowledge gaps among young couples	NA	• 97% (34 of 35) participants were completely satisfied/somewhat satisfied with TARANG intervention • 100% (35 of 35) participants will very likely recommend a friend to join the TARANG intervention	Confirmation of alignment between a priori indicator in quantitative findings and qualitative reports from participants and staff
Usefulness of the intervention defined as proportion of participants who found the TARANG intervention sessions useful or somewhat useful	The TARANG intervention proved beneficial across multiple dimensions, including enhanced family planning knowledge, increased autonomy, improved decision-making abilities, and stronger relationships with family members Participants particularly valued the social connections fostered through the program. As one participant explained: “We make friends by attending the meeting… All my friends are those who attend the meeting. As we get to know each other, we can laugh and joke together, and talk with each other.”	NGO staff and moderators emphasized TARANG's significant impact on addressing critical knowledge gaps among newly married women. Prior to the intervention, participants lacked basic understanding about reproductive health, including pregnancy and contraception. As one moderator explained: “They are becoming aware of health. When we conducted sessions, they didn't know before how pregnancy occurs… Through activities, they are getting this kind of information… They now understand menstruation, are aware of their health, and know the benefit of spacing in children, including family planning awareness.” This feedback highlighted the program’s crucial role in providing essential health education through engaging, activity-based learning methods	NA	• All participants (35 of 35) found the sessions useful and applicable to daily life • Most participants (30 of 35 or 85.7%) reported increased confidence in decision-making about childbearing and health • All participants (35 of 35) experienced strong connections with other participants and positive changes in family planning decisions • Nearly all participants (34 of 35 or 97.1%) reported improved communication with husbands about family planning Almost all participants found the session format appropriate, with 97.1%(34 of 35 participants) agreeing that both the interval between sessions and number of sessions were "just right"	Confirmation of alignment between outcomes in quantitative findings and qualitative reports from participants and staff
Improvement to the program	Participants expressed high satisfaction with TARANG’s content and delivery, particularly praising moderators’ engaging presentation style. They also supported incorporating videos to enhance learning, as one 23-year-old participant noted: “Watching videos will help in understanding, it's like a story only.”	Moderators endorsed the intervention's content while suggesting specific improvements: longer sessions to accommodate personal sharing, video integration, and additional rapport-building opportunities. As one moderator explained: “If the session duration is increased… During the session, participants also share personal experiences, so it's important to listen to them too.”	NA	• 100% felt session length was appropriate • 97.1% agreed both session intervals and number of sessions were suitable	There were some discrepancies between the quantitative results and some of the qualitative data in terms of program improvements

## Discussion

To our knowledge, this is the first mixed methods study to assess the feasibility of a life skills’ and reproductive health empowerment intervention among newly married women in rural communities in low-and-middle-income countries. The aligned quantitative and qualitative findings suggested that the 16-week intervention, conducted over six months, was acceptable, feasible, and beneficial to participants.

The quantitative results from our feasibility and acceptability assessment indicate strong support for the TARANG intervention among participants, with over 80% of participants attending half the sessions and reporting high satisfaction. All participants found the intervention useful and were willing to recommend it to others. NGO staff endorsed the program’s community acceptance and necessity for newly married women. This is especially crucial since past studies have had challenges in recruiting and retaining newly married women in similar populations [37]. While we do not have comparable studies from India or even South Asia, our results are especially promising given that no intervention to date from India has specifically targeted the sexual reproductive health needs or skill building of young newly married women.

Complementing these quantitative findings, the qualitative analysis revealed participants’ appreciation for the opportunity to engage in open discussions about sensitive topics such as family planning and menstruation, while building supportive community networks. The intervention was perceived as filling a crucial gap in knowledge, providing practical information that was previously unavailable to participants. Participants reported gaining new knowledge about contraception and reproductive health, increased decision-making autonomy, and improved communication within marital and familial relationships, highlighting the intervention’s holistic impact beyond knowledge acquisition. Access to comprehensive sexual and reproductive health knowledge empowers young women to make informed decisions about family planning, plan their families better, leading to improved maternal and child health outcomes [55].

Despite a positive reception, qualitative analysis revealed several participation barriers: recruitment challenges, women’s busy schedules, logistical constraints, cultural practices of extended stays at natal homes, festivals, household duties, and the need for rapport building. These challenges, consistent with findings from other women’s group studies [28], led to intervention refinements for the main trial to enhance participant engagement.

Based on the integrated participant and NGO staff feedback reported here, several key modifications were made to the intervention for the larger cluster randomized controlled trial. Changes included reducing sessions from 16 to 14 while increasing rapport-building sessions (1 to 2), introducing participants to local health workers in the first session, and allowing flexible scheduling with at least two sessions monthly within a 5–6 month timeframe. In response to seasonal constraints such as the grass-cutting period in Rajasthan, which primarily involves women’s labor, we strategically reduced the total number of intervention sessions to complete the study within a shorter timeframe while maintaining core content delivery. This adaptation allowed us to navigate around key agricultural periods that would otherwise have significantly limited women’s participation. To accommodate varying participation patterns, we implemented a flexible attendance strategy that included supplementary make-up sessions when participation fell below 50% for any scheduled meeting. This approach ensured that participants who missed regular sessions due to agricultural work, household responsibilities, or cultural events could still access the whole intervention, thereby maintaining intervention fidelity while addressing practical constraints faced by rural women. The intervention was enhanced by incorporating educational videos in every session and varied teaching methods including pictures, stories, and case studies. Session duration was extended to 75 min to accommodate participants’ desire for deeper discussions. The new session schedule with enhanced content targets women’s agency, reproductive autonomy, negotiation, and spousal communication (see Additional file 2). The revised intervention program enhances the pilot through strategic sequencing of financial literacy earlier with direct connections to family planning; integration of previously separated topics; practical spousal communication and negotiation training; community health worker involvement; strengthened content on consent, negotiation, and violence prevention; comprehensive coverage of planned and unplanned pregnancies; attention to sexual wellbeing; and concrete family planning decision-making in the final session.

### Strengths and limitations

This pilot study provided valuable insights for future reproductive health interventions in rural contexts, despite several limitations. Key limitations included: the restricted recruitment pool of newly married women in the study area, resulting in a small sample of 42 participants across four villages in one district of India; absence of feedback from the six participants who did not attend any sessions (a significant missed opportunity to understand barriers to engagement); and recruitment rate reporting constraints due to incomplete household listing. Finally, we did not set pre-specified progression criteria to determine whether to proceed to a definitive trial, which is recommended best practice for feasibility studies [56, 57]. Future feasibility studies should establish explicit ‘stop/go’ criteria during protocol development to enhance objectivity in trial progression decisions [58].

The study’s strengths included its mixed-methods approach (combining surveys, interviews, and monitoring data). This pilot successfully demonstrated intervention feasibility and acceptability in rural/tribal Southern Rajasthan, providing crucial groundwork for a forthcoming larger-scale randomized controlled trial and contributing to broader implementation science.

## Conclusion

The pilot study of TARANG, a reproductive health and life skills intervention, demonstrated high feasibility and acceptability among newly married women in rural/tribal Rajasthan. Mixed methods findings revealed strong participant satisfaction and positive impacts on reproductive health knowledge, communication, social connections, and empowerment, while implementation learnings informed key adaptations for the upcoming randomized controlled trial.

## Supplementary Information

Additional file 1: Appendix 2 : Sessions covered in pilot and revised intervention for RCT.

Additional file 2: RCT for Tarang Intervention: Pilot Endline Survey for Daughter-in-Law.

Additional file 3: Interview Guides for Newly Married Women and NGO staff/moderators.

## Data Availability

The quantitative data that support the findings of this study are available on request from the corresponding author. The qualitative data, given the small sample size, are not available to protect the identity of participants.

## Footnotes

1 Due to the sensitive nature of reproductive health discussions in close-knit rural communities and small sample size per village, we have not named villages.

2 The caste system is a hierarchical social stratification structure in India with historical implications for social status and opportunity. The Government of India officially classifies castes into administrative categories for affirmative action policies: Scheduled Tribes (indigenous communities), Scheduled Castes (historically most marginalized groups), Other Backward Classes (socioeconomically disadvantaged groups), and General Category (not eligible for caste-based affirmative action). These classifications correlate with documented disparities in health access and outcomes.

## References

[1] International Institute for Population Sciences (IIPS) and ICF. National Family Health Survey (NFHS-5), 2019–21. Demographic and Health Survey. Mumbai: International Institute for Population Sciences; 2021.

[2] Jones GW. Changing marriage patterns in Asia. In: Routledge Handbook of Asian Demography. Routledge; 2017.

[3] Allendorf K, Pandian RK. The decline of arranged marriage? Marital change and continuity in India. Popul Dev Rev. 2016;42:435–64. 10.1111/j.1728-4457.2016.00149.x. 28344368 10.1111/j.1728-4457.2016.00149.x PMC5362258

[4] Khalil U, Mookerjee S. Patrilocal residence and women’s social status: evidence from South Asia. Econ Dev Cult Change. 2019. 10.1086/697584.

[5] Gopalakrishnan L, Bertozzi S, Rabe-Hesketh S. Role of marriage, motherhood, son preference on adolescent girls’ and young women’s empowerment: evidence from a panel study in India. PLoS One. 2023;18:e0292084. 10.1371/journal.pone.0292084. 37769003 10.1371/journal.pone.0292084 PMC10538655

[6] Skordis J, Pace N, Vera-Hernandez M, Rasul I, Fitzsimons E, Osrin D, et al. Family networks and healthy behaviour: evidence from Nepal. Health Econ Policy Law. 2019;14:231–48. 10.1017/S1744133118000130. 29785890 10.1017/S1744133118000130

[7] Anukriti S, Herrera-Almanza C, Pathak PK, Karra M. Curse of the mummy-ji: the influence of mothers-in-law on women in India†. Am J Agric Econ. 2020;102:1328–51. 10.1111/ajae.12114.

[8] Dixit A, Bhan N, Benmarhnia T, Reed E, Kiene SM, Silverman J, et al. The association between early in marriage fertility pressure from in-laws’ and family planning behaviors, among married adolescent girls in Bihar and Uttar Pradesh, India. Reprod Health. 2021;18:60. 10.1186/s12978-021-01116-9. 33750403 10.1186/s12978-021-01116-9 PMC7941884

[9] Ghule M, Raj A, Palaye P, Dasgupta A, Nair S, Saggurti N, et al. Barriers to use contraceptive methods among rural young married couples in Maharashtra, India: qualitative findings. Asian J Res Soc Sci Humanit. 2015;5:18–33. 10.5958/2249-7315.2015.00132.X. 29430437 10.5958/2249-7315.2015.00132.X PMC5802376

[10] Dixit Anvita, Ghule Mohan, Rao Namratha, Battala Madhusudana, Begum Shahina, Johns Nicole E., et al. Qualitative examination of the role and influence of mothers-in-law on young married couples’ family planning in rural Maharashtra, India. Glob Health Sci Pract. 2022. 10.9745/ghsp-d-22-00050. 36316150 10.9745/GHSP-D-22-00050 PMC9622279

[11] Mukherjee S, Mahapatra B, Saggurti N. Why women do not use contraceptives: exploring the role of male out-migration. PLoS One. 2021;16:e0249177. 10.1371/journal.pone.0249177. 33784370 10.1371/journal.pone.0249177 PMC8009410

[12] Jain M, Caplan Y, Ramesh BM, Isac S, Anand P, Engl E, et al. Understanding drivers of family planning in rural northern India: an integrated mixed-methods approach. PLoS One. 2021;16:e0243854. 10.1371/journal.pone.0243854. 33439888 10.1371/journal.pone.0243854 PMC7806122

[13] Banerjee SK, Andersen KL, Warvadekar J, Aich P, Rawat A, Upadhyay B. How prepared are young, rural women in India to address their sexual and reproductive health needs? A cross-sectional assessment of youth in Jharkhand. Reprod Health. 2015;12:97. 10.1186/s12978-015-0086-8. 26476778 10.1186/s12978-015-0086-8 PMC4609062

[14] Rose-Clarke K, Pradhan H, Rath S, Rath S, Samal S, Gagrai S, et al. Adolescent girls’ health, nutrition and wellbeing in rural eastern India: a descriptive, cross-sectional community-based study. BMC Public Health. 2019;19:673. 10.1186/s12889-019-7053-1. 31151394 10.1186/s12889-019-7053-1 PMC6544920

[15] McClendon KA, McDougal L, Ayyaluru S, Belayneh Y, Sinha A, Silverman JG, et al. Intersections of girl child marriage and family planning beliefs and use: qualitative findings from Ethiopia and India. Cult Health Sex. 2018;20:799–814. 10.1080/13691058.2017.1383513. 29043910 10.1080/13691058.2017.1383513

[16] Ghimire A, Samuels F. Change and continuity in social norms and practices around marriage and education in Nepal. ODI; 2014.

[17] Kumar P, Nuken A, Datta N, Vyas A. Impact of an empowerment and employability program for adolescent girls: evidence from India. J Youth Dev. 2021;16:255–77. 10.5195/jyd.2021.1048.

[18] Majumdar P, Purkayastha S, Goswami D. Empowerment of adolescent girls and gender based violence prevention through sports: a group work intervention in India. Soc Work Groups. 2023;46:338–48. 10.1080/01609513.2022.2124495.

[19] Dhar D, Jain T, Jayachandran S. Reshaping adolescents’ gender attitudes: evidence from a school-based experiment in India. Am Econ Rev. 2022;112:899–927. 10.1257/aer.20201112.

[20] Houweling TA, Tripathy P, Nair N, Rath S, Rath S, Gope R, et al. The equity impact of participatory women’s groups to reduce neonatal mortality in India: secondary analysis of a cluster-randomised trial. Int J Epidemiol. 2013;42:520–32. 10.1093/ije/dyt012. 23509239 10.1093/ije/dyt012 PMC3619953

[21] Gope RK, Tripathy P, Prasad V, Pradhan H, Sinha RK, Panda R, et al. Effects of participatory learning and action with women’s groups, counselling through home visits and crèches on undernutrition among children under three years in eastern India: a quasi-experimental study. BMC Public Health. 2019;19:962. 10.1186/s12889-019-7274-3. 31319828 10.1186/s12889-019-7274-3 PMC6637592

[22] Tripathy P, Nair N, Sinha R, Rath S, Gope RK, Rath S, et al. Effect of participatory women’s groups facilitated by accredited social health activists on birth outcomes in rural eastern India: a cluster-randomised controlled trial. Lancet Glob Health. 2016;4:e119–28. 10.1016/S2214-109X(15)00287-9. 26823213 10.1016/S2214-109X(15)00287-9

[23] Shah H, Adyanthaya S, Gope RK, Kumar A. Using the participatory learning and action approach to improve community engagement: learnings from India. 2023.

[24] Ministry of Rural Development G of I. Dashboard-NRLM eGov application-government of India. Dashborad of National Rural Health Mission, Inid. https://nrlm.gov.in/dashboardForOuter.do?methodName=dashboard#. Accessed 28 Mar 2024.

[25] Saggurti N, Atmavilas Y, Porwal A, Schooley J, Das R, Kande N, et al. Effect of health intervention integration within women’s self-help groups on collectivization and healthy practices around reproductive, maternal, neonatal and child health in rural India. PLoS One. 2018;13:e0202562. 10.1371/journal.pone.0202562. 30138397 10.1371/journal.pone.0202562 PMC6107172

[26] Kumar A, Sethi V, de Wagt A, Parhi RN, Bhattacharjee S, Unisa S, et al. Evaluation of impact of engaging federations of women groups to improve women’s nutrition interventions- before, during and after pregnancy in social and economically backward geographies: evidence from three eastern Indian States. PLoS One. 2023;18:e0291866. 10.1371/journal.pone.0291866. 37797057 10.1371/journal.pone.0291866 PMC10553280

[27] Chandrashekar S, Saha S, Varghese B, Mohan L, Shetty G, Porwal A, et al. Cost and cost-effectiveness of health behavior change interventions implemented with self-help groups in Bihar, India. PLoS One. 2019;14:e0213723. 10.1371/journal.pone.0213723. 30921334 10.1371/journal.pone.0213723 PMC6438566

[28] Desai S, Misra M, Das A, Singh RJ, Sehgal M, Gram L, et al. Community interventions with women’s groups to improve women’s and children’s health in India: a mixed-methods systematic review of effects, enablers and barriers. BMJ Glob Health. 2020;5:e003304. 10.1136/bmjgh-2020-003304. 33328199 10.1136/bmjgh-2020-003304 PMC7745316

[29] Patton GC, Sawyer SM, Santelli JS, Ross DA, Afifi R, Allen NB, et al. Our future: a lancet commission on adolescent health and wellbeing. Lancet. 2016;387:2423–78. 10.1016/S0140-6736(16)00579-1. 27174304 10.1016/S0140-6736(16)00579-1 PMC5832967

[30] UNICEF. Global Evaluation of Life Skills Education Programmes Evaluation. New York; 2012.

[31] UNICEF. Review of the Life Skills Education Program. Maldives; 2016.

[32] UNICEF, UNFPA. Technical note on life skills programmes for empowering adolescent girls: notes for practitioners on what works. 2019.

[33] Svanemyr J, Baig Q, Chandra-Mouli V. Scaling up of life skills based education in Pakistan: a case study. Sex Educ. 2015;15:249–62. 10.1080/14681811.2014.1000454.

[34] Kalanda BF. Life skills and reproductive health education changes behaviour in students and teachers: evidence from Malawi. Educational Research and Reviews. 2010;5:169–74. https://doi.org/10/Apr/Kalanda.pdf.

[35] Singla DR, Waqas A, Hamdani SU, Suleman N, Zafar SW, Zill-e-Huma, et al. Implementation and effectiveness of adolescent life skills programs in low- and middle-income countries: a critical review and meta-analysis. Behav Res Ther. 2020;130:103402. 10.1016/j.brat.2019.04.010. 10.1016/j.brat.2019.04.010 31146889

[36] Atif N, Rahman A, Huma Z, Hamdani SU. Preparing for parenthood: developing a life-skills and socioemotional health program for young married couples in rural Pakistan. Glob Health Action. 2021;14:1982485. 10.1080/16549716.2021.1982485. 34605368 10.1080/16549716.2021.1982485 PMC8491718

[37] Sharma S, Soni R, Achyut P, Das M, Verma R, Gautam A, et al. Promoting adolescent engagement, knowledge and health (PAnKH) in Rajasthan, India. The IFS; 2019. 10.1920/re.ifs.2019.0161.

[38] Raj A, Ghule M, Johns NE, Battala M, Begum S, Dixit A, et al. Evaluation of a gender synchronized family planning intervention for married couples in rural India: the CHARM2 cluster randomized control trial. eClinicalMedicine. 2022. 10.1016/j.eclinm.2022.101334. 35274093 10.1016/j.eclinm.2022.101334 PMC8902598

[39] Santhya KG, Haberland N, Das A, Ram F, Sinha RK, Ram U, et al. Empowering married young women and improving their sexual and reproductive health: Effects of the First-time Parents Project. Poverty, Gender, and Youth. 2008. 10.31899/pgy5.1005.

[40] Raj A, Ghule M, Ritter J, Battala M, Gajanan V, Nair S, et al. Cluster randomized controlled trial evaluation of a gender equity and family planning intervention for married men and couples in rural India. PLoS One. 2016;11:e0153190. 10.1371/journal.pone.0153190. 27167981 10.1371/journal.pone.0153190 PMC4864357

[41] Raj A, Ghule M, Johns N, Battala M, Begum S, Averbach S. CHARM 2: a gender synchronized family planning intervention for couples in rural India, a cluster randomized trial [A05]. Obstet Gynecol. 2022;139:2S-2S. 10.1097/01.aog.0000826356.93844.ca.

[42] Subramanian L, Simon C, Daniel EE. Increasing contraceptive use among young married couples in Bihar, India: evidence from a decade of implementation of the PRACHAR project. Glob Health Sci Pract. 2018;6:330–44. 10.9745/GHSP-D-17-00440. 29959273 10.9745/GHSP-D-17-00440 PMC6024625

[43] Diamond-Smith N, Gopalakrishnan L, Hannah L, Elizabeth K, Cynthia H, Sheri W, et al. A life skills and reproductive health empowerment intervention for newly married women and their families to reduce unintended pregnancy in India: Protocol for the TARANG cluster randomized controlled trial. 2024. 10.1136/bmjopen-2024-086778 PMC11086273 38688674

[44] Diamond-Smith N, Vaishnav Y, Choudhary U, Sharma P, Kachhwaha A, Panjalingam T, et al. Individual empowerment and community norm effects of engaging young husbands in reproductive health in rural India: findings from a pilot study. 2024. 10.21203/rs.3.rs-4376443/v1. 10.1186/s12978-024-01878-y PMC11488357 39420379

[45] National Family Health Survey (NFHS-5) India 2019–21. 2022.

[46] StataCorp,. Stata Statistical Software: Release 15. 2017.

[47] Cocks K, Torgerson DJ. Sample size calculations for pilot randomized trials: a confidence interval approach. J Clin Epidemiol. 2013;66:197–201. 10.1016/j.jclinepi.2012.09.002. 23195919 10.1016/j.jclinepi.2012.09.002

[48] Sim J, Lewis M. The size of a pilot study for a clinical trial should be calculated in relation to considerations of precision and efficiency. J Clin Epidemiol. 2012;65:301–8. 10.1016/j.jclinepi.2011.07.011. 22169081 10.1016/j.jclinepi.2011.07.011

[49] Guest G, Namey E, Chen M. A simple method to assess and report thematic saturation in qualitative research. PLoS One. 2020;15:e0232076. 10.1371/journal.pone.0232076. 32369511 10.1371/journal.pone.0232076 PMC7200005

[50] Dedoose Version 9.0.17, web application for managing, analyzing, and presenting qualitative and mixed method research data (2021).

[51] Braun V, Clarke V. Using thematic analysis in psychology. Qual Res Psychol. 2006;3:77–101. 10.1191/1478088706qp063oa.

[52] Creswell JW, Creswell JD. Research design: qualitative, quantitative, and mixed methods approaches. SAGE Publications; 2017.

[53] Fetters MD, Curry LA, Creswell JW. Achieving integration in mixed methods designs—principles and practices. Health Serv Res. 2013;48:2134–56. 10.1111/1475-6773.12117. 24279835 10.1111/1475-6773.12117 PMC4097839

[54] Fetters MD, Molina-Azorin JF. Utilizing a mixed methods approach for conducting interventional evaluations. J Mixed Methods Res. 2020;14:131–44. 10.1177/1558689820912856.

[55] Santhya KG, Jejeebhoy SJ. Sexual and reproductive health and rights of adolescent girls: evidence from low- and middle-income countries. Glob Public Health. 2015;10:189–221. 10.1080/17441692.2014.986169. 25554828 10.1080/17441692.2014.986169 PMC4318087

[56] Eldridge SM, Chan CL, Campbell MJ, Bond CM, Hopewell S, Thabane L, et al. CONSORT 2010 statement: extension to randomised pilot and feasibility trials. BMJ. 2016;355:i5239. 10.1136/bmj.i5239. 27777223 10.1136/bmj.i5239 PMC5076380

[57] Avery KNL, Williamson PR, Gamble C, Francischetto EO, Metcalfe C, Davidson P, et al. Informing efficient randomised controlled trials: exploration of challenges in developing progression criteria for internal pilot studies. BMJ Open. 2017;7:e013537. 10.1136/bmjopen-2016-013537. 28213598 10.1136/bmjopen-2016-013537 PMC5318608

[58] Bugge C, Williams B, Hagen S, Logan J, Glazener C, Pringle S, et al. A process for decision-making after pilot and feasibility trials (ADePT): development following a feasibility study of a complex intervention for pelvic organ prolapse. Trials. 2013;14:353. 10.1186/1745-6215-14-353. 24160371 10.1186/1745-6215-14-353 PMC3819659

