# Supplementary material for: Feasibility and acceptability of a life skills and reproductive health empowerment intervention for young newly married women in Rajasthan, India: a pre-post convergent mixed methods pilot study
Source: Pilot Feasibility Stud. 2025 Nov 15;11:142. doi: 10.1186/s40814-025-01720-7 (PMC12619424; doi:10.1186/s40814-025-01720-7)
Supplement: Supplementary file 1 — Additional file 1: Appendix 2 : Sessions covered in pilot and revised intervention for RCT. [file 40814_2025_1720_MOESM1_ESM.docx]

**Appendix 2: Sessions covered in pilot and revised intervention for RCT**

| **Pilot** | | **Revised Intervention for RCT** | |
| --- | --- | --- | --- |
| Session | Name of the session | Session | Name of the session |
| Session 1: | My Health My priority | Session 1: | Forming the group |
| Session 2: | Know your body | Session 2: | Kick Start |
| Session 3: | Science behind conception | Session 3: | Language of Money |
| Session 4: | Contraception Methods | Session 4: | Contraception methods |
| Session 5: | Love has no space for violence | Session 5: | How to choose family planning methods |
| Session 6: | My Identity | Session 6: | My Health My priority |
| Session 7: | Responsible sexual behavior | Session 7 | Know your body |
| Session 8: | My consent is import! | Session 8: | Responsible sexual behavior |
| Session 9: | How to choose family planning methods | Session 9: | My consent is import! |
| Session 10: | Negotiate your way | Session 10: | Science behind conception |
| Session 11: | Language of Money | Session 11: | Negotiate your way |
| Session 12: | Preparing for digital world | Session 12: | Safe maternity, healthy life |
| Session 13: | My five-year plan | Session 13: | Love has no space for violence |
| Session 14: | Conception and Abortion | Session 14: | My five-year plan |
| Session 15: | Safe maternity, healthy life |  |  |
| Session 16: | Managing emotions and dealing with stress |  |  |

**Side-by-Side Comparison of Pilot and Revised Intervention for Newly Married Women**

| **Aspect** | **Pilot Program** | **Revised Intervention for RCT** | **Key Differences** |
| --- | --- | --- | --- |
| **Number of Sessions** | 16 sessions | 14 sessions | Intervention is more condensed |
| **Introductory Sessions** | Session 0: Kick Start | Sessions 1-2: Mobilization, rapport building, and kick start | Intervention places stronger emphasis on rapport building and community support systems |
| **Financial Literacy** | Session 11: Language of Money | Session 3: Language of Money | Financial literacy prioritized much earlier in intervention; explicitly connected to family planning readiness |
| **Contraception** | Session 4: Contraception Methods | Session 4: Contraception methods + medication abortion + invite ASHA | Intervention combines contraception with abortion information and includes community health worker (ASHA) |
| **Family Planning** | Session 9: How to choose family planning methods | Session 5: How to choose family planning methods (with spousal communication practice) | Intervention adds practical communication skills component |
| **Health Awareness** | Session 1: My Health My priority | Session 6: My Health, my priority (with identity exploration) | Intervention integrates personal identity with health awareness |
| **Body Knowledge** | Session 2: Know your body | Session 7: Know your body | Similar focus in both programs |
| **Sexual Health** | Sessions 2, 4, 7, 13, 14, 15 (scattered) | Session 8: Sexual Wellbeing + pleasure + STD/STI | Intervention creates dedicated session on sexual wellbeing and pleasure |
| **Consent** | Session 8: My consent is import! | Session 9: My consent is import! (with time sensitivity emphasized) | Intervention clarifies the ongoing nature of consent |
| **Conception Science** | Session 3: Science behind conception | Session 10: Science behind conception | Similar positioning in sequence |
| **Negotiation Skills** | Session 10: Negotiate your way | Session 11: Negotiate your way (with spousal FP communication) | Intervention explicitly focuses on family planning communication |
| **Pregnancy Planning** | Session 14: Conception and Abortion | Session 12: Preparing for pregnancy + dealing with abortion | Intervention frames both planned and unplanned pregnancy more holistically |
| **Violence Prevention** | Session 5: Love has no space for violence | Session 13: Love has no space for violence | Intervention places this later, after building other skills |
| **Future Planning** | Session 13: My five-year plan | Session 14: My five-year plan | Both programs conclude with planning, but intervention specifically cementing family planning decisions |

**Key Enhancements in the Intervention Program**

1. **Strategic Sequencing and Reduced sessions**: The intervention program resequences content to build skills progressively, placing financial literacy earlier and connecting it directly to family planning decisions. The main study curriculum was condensed from a total of 16 sessions to 14 sessions.
2. **Integrated Approach**: The intervention combines related topics that were separated in the pilot (e.g., contraception and abortion; health awareness and identity). Content on 'abortion' and myths associated with abortion were integrated into the "Contraception methods" session. This led to removing the separate "Conception and Abortion" session.
3. **Removal of two sessions**: "Preparing for digital world" (digital literacy) and "Managing emotions and dealing with stress" (emotion regulation).
4. **Practical Communication Focus**: The intervention emphasizes practicing actual communication with spouses about family planning choices. The "My Identity" session was removed, and its key messages were integrated with "My Health My priority" session.
5. **Community Connection**: The intervention explicitly includes community health workers (ASHA) and establishes connections to the village support structure.
6. **Enhanced Agency Elements**: The intervention strengthens content related to consent, negotiation, and responding to intimate partner violence.
7. **Comprehensive Reproductive Planning**: The intervention addresses both planned and unplanned pregnancy scenarios in an integrated manner.
8. **Sexual Wellbeing**: The intervention includes specific content on sexual pleasure, moving beyond just reproductive health.
9. **Clear Decision Outcomes**: The intervention concludes with concrete family planning decisions, whereas the pilot’s final session was more general.
