# Supplementary material for: Feasibility and acceptability of a life skills and reproductive health empowerment intervention for young newly married women in Rajasthan, India: a pre-post convergent mixed methods pilot study
Source: Pilot Feasibility Stud. 2025 Nov 15;11:142. doi: 10.1186/s40814-025-01720-7 (PMC12619424; doi:10.1186/s40814-025-01720-7)
Supplement: Supplementary file 2 — Additional file 2: RCT for Tarang Intervention: Pilot Endline Survey for Daughter-in-Law. [file 40814_2025_1720_MOESM2_ESM.docx]

**RCT for Tarang Intervention**

**Pilot Endline Survey for Daughter-in-Law**

**Date updated: 8^th^ January, 24**

INFORMED CONSENT FORM (to be placed)

Contents

[SECTION A: ADMINISTRATIVE 2](#_Toc153549142)

[SECTION B: DEMOGRAPHICS 4](#_Toc153549143)

[SECTION C: FERTILITY PREFERENCES AND DECISION-MAKING 7](#_Toc153549144)

[SECTION D: USE OF FAMILY PLANNING METHODS 9](#_Toc153549145)

[SECTION E: PREGNANCY AND BIRTH HISTORY 11](#_Toc153549146)

[SECTION F: WOMEN’S EMPOWERMENT 14](#_Toc153549147)

[SECTION G: RELATIONSHIP QUALITY 20](#_Toc153549148)

[SECTION H: ASPIRATIONS AND HOPE 22](#_Toc153549149)

[SECTION I: BELIEFS AND NORMS 23](#_Toc153549150)

[SECTION J: ANXIETY AND DEPRESSION 26](#_Toc153549151)

[SECTION K: KNOWLEDGE 26](#_Toc153549152)

[SECTION L: TIME USE 29](#_Toc153549153)

[Section M: TARANG INTERVENTION MEASURES 31](#_Toc153549154)

# SECTION A: ADMINISTRATIVE

| **Survey date**: _______________________ *CAPI will auto-calculate.* |
| --- |
| A1. District Name: ______________________________ b. Code   *(CAPI: Provide drop down menu to select.)*  *Deidentified: Only codes will be provided.* |
| A2. Name of the Block: __________________________ a.Code   *(CAPI: Provide drop down menu to select.)*  *Deidentified: Only codes will be provided.* |
| A3. Name of the Village: __________________________ a.Code   *(CAPI: Provide drop down menu to select.)*  *Deidentified: Only codes will be provided.* |
| **A4. Name of the DIL (Age, Unique ID)**  *CAPI: Show drop-down list of recruited households for enumerators to select one (For DIL interview)*  *Deidentified: Only unique ID will be provided.* |
| **A5. Interviewer: Please write down the HH ID from the control sheet.**  Control Sheet HH ID **________________** |
| Interviewer: You have selected the following house. Please confirm and then proceed.  *CAPI: On the next screen show all the house identification details like:*  In the next screen show all identifiable details of the household such as  Name of the head  Name of DIL and age  Name of Husband and age  Name of MIL and age  Address and phone number of the household  *CAPI ID: Will be autogenerated.*  *Note this CAPI ID in your contact recorder.*  *Deidentified: Names, Addr., Phone no. and CAPI ID won’t be provided* |
| A6. Enumerator: Please confirm the above information is correct and you are visiting the right HH?  1. Yes 0. No  *(CAPI: Only if coded “1.Yes” allow to go further. If coded “0.No” display message “Kindly inform your Supervisor and select the correct HH ID)* |
| A7 (a). Name of the interviewer: ___________________________. b Code   *(CAPI: Display interviewers names via drop down menu for selection and pull the respective ID and Name.)*  *Deidentified: Only interviewer ID will be provided.* |
| **A8. Is Survey of the respondent possible?**   1. Yes *(CAPI: Continue the interview)* 2. Respondent not at home despite repeated visits *(CAPI: Capture GPS and end the interview)* 3. Household not found. *(CAPI: Capture GPS and end the interview)* |
| A9(a). Start time: _____________  A9(b) End time: _____________  *CAPI: Record it at the backend of CAPI* |
| ***A10. Administer Consent for the daughter-in-law-* do you agree to participate in this study?**  1. Respondent agreed to participate in the survey (Continue the interview)  0. No, consent refused/denied (Capture GPS and end the interview)  Interviewer: If “yes” then please give printed consent form to the respondent for his records.  *CAPI: Insert the feature to get respondent’s Signature if “1. Yes” been coded.* |
| **A12. Interviewer: Is the respondent willing to give signature consent.**   1. Yes, agreed for the signature. 2. No, agreed for audio recording. |
| ***Ask if 12 = 1***  **A12a. Interviewer: Please obtain the signature of the respondent.**  *(CAPI: Insert the feature to get respondent’s signature.)*  *Deidentified* |
| ***Ask if 12 = 2***  **A12c. Interviewer: Please ask the respondent to read aloud –**  "Yes, I agree to participate in the survey.”  *(CAPI: Insert the feature to get respondent’s signature.)*  *Deidentified* |
| **A13. Capture GPS location**  Stop for 15-20 seconds and try 2-3 times at home or in an open place near home.  *Deidentified* |
| A14. Result of the interview:  1. Interview completed  2. Partially completed (return visit not possible)  3. Respondent not at home despite repeated visits  4. Household not found  5. Respondent Refused Participation / Did not consent  6. Mother-in-law / household head / husband refused respondents participation  7. Daughter-in-law is found to be underage / interview suspended  77. Moved from in laws place due to some issue/conflict  999. The respondent was identified as migrant during baseline |
| **A15. Did anyone do spot checks or accompany check for this survey?**  0. No  1. Spot check  2. Full accompanied call |
| **A16. Who conducted SC or AC?**  1. Team Supervisor  2. Field Manager / Executive  3. Researchers  4. Clients/partners  *Deidentified* |
| A17. **Name of the person conducting AC or SC?**  ____________________________  *Deidentified* |
| A18. **14. Interviewer’s Team Id:**  ____________________________ |
| A19. **Write notes for researchers / Data team**  ____________________________ |

# SECTION C: FERTILITY PREFERENCES AND DECISION-MAKING

**READ: Now I have some questions about your preferences and decisions about having children in the future.**

| **Q. N** | **Questions** | **Coding Categories** |  |
| --- | --- | --- | --- |
| **C1** | What would be the ideal number of total children you would like to have? | _______ **(Range: 0-10)**   1. God’s will/Depends.   -99 Don’t know | |
| **C2** | What is your ideal gap between your marriage and the birth of the first child in months?  *(Hint-If she says already pregnant, then ask how much gap she would have like to keep)* | \|__\|__\| months **(Range: 0-60)**  **Enter:**  0 if says immediately.  -77 More than 60 months (more than 5 years)  -99 if don’t know / no preference | |
| **C3** | ***Ask if C2 ≠ -99***  What are the reasons you would like to keep (XXX from C2) months of gap from now and the first pregnancy?  *CAPI- Estimate xxx days from C2.*  **Multiple Response possible** | 1. In-laws want to have child with this gap  2. I want to have child with this gap  3. It will be good for the health of child/me to keep this gap  5. It will improve relationship with my husband/in-laws  7. I am told this is the right gap to keep by relatives/health care provider  8. Need to prove fertility  9. Standing in family will increase if I have child with this gap  10. This gap will help me continue my education  11. This gap will help me be able to work/earn  12. We cannot afford a child at this time  13; I want to get to know my husband better  14. Husband wants to have children with this gap.  15. Due to society’s thoughts/perception95. Others (Specify _______)  -99. No specific reason / Don't know | |
| **C4** | According to you, how many months gap would you like to keep between your first and second child? | \|__\|__\| months ***(Range: 0-120)***  **Enter:**  -77 More than 120 months (more than 10 years)-97. Whenever God wants  -99 if don’t know / no preference | |
| **C5** | Imagine that would have a total of three children, what would you like the most – a boy or a girl? How many boys and how many girls?  .  *(Hint- Imagine the situation)* | 0. No preference/ I am okay whatever happens.  1. Three girls  2. One boy and two girls  3. Two boys and one girl  4. Three boys  -99. Don’t know/cannot say. | |

**READ: Now I am going to ask you questions about discussions and decision making about having children from the time you got married until now.**

| **Q. N** | **Questions** | **Coding Categories** |
| --- | --- | --- |
| **C6** | Have you and your husband **ever discussed** the following topics together? | 0. No  1. Yes |
| a | How many children you should have? | 0 1 |
| b | When should you have the first child? | 0 1 |
| c | How much gap should be kept between children? | 0 1 |
| d | Whether and which family planning methods to use? | 0 1 |
| **C7** | Was **any decision made** in this household about the following?  *(Hint-By anyone whether the couple or in-laws)* | 0. No  1. Yes  -99 Don’t know |
| a | How many children you should have? | 0 1 -99 |
| b | When should you have the first child? | 0 1 -99 |
| c | How much gap should be kept between children? | 0 1 -99 |
| d | Whether and which family planning methods to use? | 0 1 -99 |
| **C8** | ***Ask if C7 = 1 for a-d respectively***  Who **mainly made the decision** about the following? | 1 Respondent (Wife)  2 Husband  3 Respondent and husband jointly  4 Others |
| a | How many children you should have? | 1 2 3 4 |
| b | When should you have the first child? | 1 2 3 4 |
| c | How much gap should be kept between children? | 1 2 3 4 |
| d | Whether and which family planning methods to use? | 1 2 3 4 |
| **C9** | ***Ask if C8 = 2 or 4 for a-d respectively***  Did you feel like **your opinion was considered in deciding** about the following? | 0. No  1. Yes |
| a | How many children you should have? | 0 1 |
| b | When should you have the first child? | 0 1 |
| c | How much gap should be kept between children? | 0 1 |
| d | Whether and which family planning methods to use? | 0 1 |

***Desire to Avoid Pregnancy***

*We would like to ask you about your current thoughts and feelings about the idea of delivering a baby in the next year and becoming the mother. People have very different thoughts and feelings about having a baby that can change over time. There are no right or wrong answers. For each statement, choose one response that seems right at this point in your life. Even if you do not think you will have a child in next 12 months, please imagine how you would feel about having a baby in next 12 months. If you are currently pregnant, then let me know what you are ac thinking or feeling at this stage.*

***(CAPI: Give ordering from strongly disagree to strongly agree and include smiley or some symbol. Note, we need to do this for all scale questions. We also need to create a picture strip to handover to the respondent with these pictures.)***

| **QN** | **Question** | **1. Strongly disagree** | **2. Disagree** | **3. cannot decide / Don’t k now** | **4. Agree** | **5. Strongly agree** |
| --- | --- | --- | --- | --- | --- | --- |
| **C10** | You want to have a baby in the next year. |  |  |  |  |  |
| **C11** | Having a baby in the next 3 months would bring you closer to your husband. |  |  |  |  |  |
| **C12** | If you had a baby in the next year, it would be bad for your life. |  |  |  |  |  |
| **C13** | Thinking about having a baby within the next year makes you smile. |  |  |  |  |  |
| **C14** | You would feel a loss of freedom if you had a baby in the next year. |  |  |  |  |  |
| **C15** | If you had a baby in the next year, it would be hard for you to manage raising the child. |  |  |  |  |  |

# SECTION D: USE OF FAMILY PLANNING METHODS

**READ: Now I would like to ask some questions about use of family planning methods. Let me assure you again that your answers are completely confidential and will not be told to anyone. If we should come to any question that you don't want to answer, just let me know and we will go to the next question.**

| **D1** | Have you consummated your marriage (had sex with husband)? | 0. No  1. Yes  -98. Refused answer |
| --- | --- | --- |
| **Now, I will ask you a few questions related to family planning** | | |
| **D2** | ***Ask if D1=1***  Have you ever or are you/your husband currently using any method to avoid or delay getting pregnancy? | 0. Never used  1. Yes, used in past but not now  2. Didn't use in the past but currently using  3. Yes, both used in the past and using currently  -98. Refused answer |
| **D3** | ***Ask if D2 = 1***  What method(s) did you/your husband use in the past?  *Multiple codes possible* | 1. Copper T  2. Antara Injections (such as Depo Provera)  3. Emergency pills (taken within 3 days)  4. Everyday pills (such as Mala-N, Chhaya)  5. Male condom  6. Female condom  7. Safe/Standard days method/ Rhythm method  8. Withdrawal  9. Female sterilization  10. Male sterilization  11. Hysterectomy  95. Other method, specify---------  -98. Refused answer |
| **D4** | ***Ask if D2 = 2 or 3***  What method(s) are you/your husband currently using?  *Multiple codes possible* | 1. Copper T  2. Antara Injection (such as Depo Provera)3. Emergency pills (taken within 3 days)  4. Everyday pills (such as Mala-N, Chhaya)  5. Male condom  6. Female condom  7. Safe/Standard days method/ Rhythm method  8. Withdrawal  9. Female sterilization  10. Male sterilization  11. Hysterectomy  95. Other method, specify---------  -98. Refused answer |
| **D5** | ***Ask if D2 = 2 or 3***  During the last month, how regularly did you/your husband use family planning methods? | 0. Not at all.  1. Sometimes  2. Most times but not all.  3. Every time  -98. Refused answer |
| **D6** | ***Ask if D2 = 0, 1 or -98***  Will you/your husband like to use a method to delay or avoid getting pregnant at any time in the future? | 0. No  1. Yes  -99 Don’t know/can’t say.  -98. Refused answer |
| **D7** | ***Ask if D6 = 1***  If you/your husband were to use a family planning method in future, what methods do you think you would use?  ***Multiple response possible*** | 1.Copper T  2. Antara Injection (such as Depo Provera)  3. Emergency pills (taken within 3 days)  4. Everyday pills (such as Mala-N, Chhaya)5. Male condom  6. Female condom  7. Safe/Standard days method/ Rhythm method  8. Withdrawal  9. Female sterilization  10. Male sterilization  11. Hysterectomy  95. Other method, specify---------  -98. Refused answer |
| **D8** | ***Ask if D6 = 1***  How many days from now do you think you will start using a method? | Days from now: _______ *(Range: 0 – 180)*  **Enter**  -77 if more than 180 days  *-*99 if don’t know / cannot say.  -98. Refused answer |
| **D9** | ***Ask if D2=0 or 1 or D6= 1***  What is your plan if your husband refuses to use or let you use contraceptive methods? | 0. I will not use any method  1. I will use a hidden/secret method like pills or copper T  2. Refuse to have sex  3. Get help of in-laws/relatives to convince him  4. I will force the use, it is my choice  5. Will explain to my husband and take him to the doctor.  95. Other  -99 Don’t know.  -98. Refused answer |

# SECTION E: PREGNANCY AND BIRTH HISTORY

**Read: Now, we will be asking you several questions about pregnancies. I also know that some of the questions may not be applicable to you or you maty be shy in answering them. But remember that any answer you give is completely confidential.**

| **QN** | **Questions** | **Codes** |
| --- | --- | --- |
| **E1a** | I recognize that this is a highly personal question, but the answer is important because frequency of sex can depend on whether a husband stays in the village or is a migrant.  How many months in the past 4 months (since August 2023) would you say your husband and you were able to establish sexual relationship? | Months ______ ***(Range: 1 – 4 months)***  **Enter:**  -99 Don’t know/can’t say.  -98 Refused answer. |
| **E2a** | ***ASK IF E1 ≠ -99 or -98***  On how many days in a typical month would you say your husband and you established sexual relationship in the past 4 months? | Days______ ***(Range: 1 – 31 days)***  **Enter:**  -99 Don’t know/can’t say.  -98 Refused answer. |
| **E3** | How many days ago did your last menstrual period start? | Days ago, ______ ***(Range: 1 – 120 days)***  **Enter:**  -99 Don’t know/can’t say.  -98 Refused answer. |
| **E4** | Are you currently pregnant? | 0. No  1. Yes  -99 Don’t know.  -98 Refused answer |
| **E6** | ***Ask if E4=0 or -99***  If you were to find that you are currently pregnant, how happy, or sad would you feel? | 1. Extremely happy  2. Sort of happy  3. Cannot decide / Don’t know  4. Sort of unhappy  5. Very unhappy  -98 Refused answer. |
| **E6b** | Did you have naturally occurring, or medically induced abortion / termination in the past 4 months? | 0. No  1. Yes  -99 Don’t know.  -98 Refused answer |

***Ask if E4=1 Now*, we will be asking a few questions about your current pregnancy**

| **E7** | ***Ask if E4=1***  When you found out you were pregnant, how did you feel? | 1. Extremely happy  2. Sort of happy  3. Cannot decide / Don’t know  4. Sort of unhappy  5. Very unhappy  -98 Refused answer. |
| --- | --- | --- |
| **E8** | ***Ask if E4=1***  At the time you became pregnant, did you want to become pregnant then, did you want to wait until later, or did you not want to have any / any more children at all? | 1. Wanted child at that time  2. Wanted child later  3. Did not want child at all  -96. Didn't think about it, just happened.  -98 Refused answer. |
| **E9** | ***Ask if E4=1***  Before you became pregnant with your current pregnancy, which best describes your situation?  *(Hint: Read responses, Select One)* | 1. You and your husband had agreed for you to get pregnant  2. You and your husband had discussed having children together but hadn’t agreed for you to get pregnant  3. You and your husband had never discussed having children together  0. None of the above  -98 Refused answer. |
| **E10** | ***Ask if E4=1***  In the month before you got pregnant (conceived), which of the following best describes your contraceptive use?  *(Hint: Read responses, Select One)* | 0. Did not use any contraception  1. Using contraception every time.  2. Were using contraception, but not on every occasion.  3. Rarely / sometimes used contraceptives  -98 Refused answer. |

**Contraceptive calendar method.** Now I will ask you for the last few months about some questions regarding your sexual engagement, contraceptive use and methods. Please try to recall for the following months and respond. Enumerators: Please fill the question in a discussion format as per your training.

| Sr No | Month | E25 | E26  *Ask if E25=1* | E27 | E28  Ask if E27=1 |
| --- | --- | --- | --- | --- | --- |
|  |  | Did your husband and you establish sexual relationship in [MONTH]  (1) Yes  (0) No | Were/are you pregnant?  (1) Yes  (0) No | Did you use any contraceptive method in [MONTH]?  (1) Yes  (0) No | What family planning method(s) did you use in [MONTH]?  (1) Semi-permanent (Copper-T, injectables)  (2) Regular methods (condom; pills; morning-after emergency pills)  (3) Traditional (Rhythm, Withdrawal)  (95) Other (specify) |
| E25_1 | DECEMBER | 0 1 | 0 1 | 0 1 | 1 2 3 95 |
| E25_2 | NOVEMBER | 0 1 | 0 1 | 0 1 | 1 2 3 95 |
| E25_3 | OCTOBER | 0 1 | 0 1 | 0 1 | 1 2 3 95 |
| E25_4 | SEPTEMBER | 0 1 | 0 1 | 0 1 | 1 2 3 95 |
| E25_5 | AUGUST | 0 1 | 0 1 | 0 1 | 1 2 3 95 |

# SECTION F: WOMEN’S EMPOWERMENT

**READ: Now we are going to ask you several questions about your bank operations, decision making in the household etc. Remember all your responses are confidential.**

| **F1** | Do you have an account in a bank, post-office or other financial institution? (In your own name or jointly with someone?) (Excludes SHG, chit fund etc.) | 0. No account  1. In Respondent’s Name  2. Jointly with someone else |
| --- | --- | --- |
| **F2** | ***Ask if F1= 1 or 2***  Do you deposit or withdraw money from this/these account(s) on your own? | 0. No  1. Yes |
| **F3** | Do you have a smart phone or feature/keypad-based mobile phone that you yourself use? | 0. No  1. Yes, smartphone  2. Yes, feature / keypad mobile  3. Both smart and feature/keyboard phone |
| **F4** | ***Ask F3 = 1, 3***  Do you use your mobile phone to send or receive money? | 0. No  1. Yes |
| **F5** | How often do you access the internet on a mobile phone or computer? Would you say almost every day, at least once a week, at least once a month, rarely or not at all? | 1. Almost everyday  2. At least once a week  3. At least once a month  4. Rarely  5. Not at all  -96. Don’t know what internet is |
| **F6b** | ***ASK IF F5 = 1, 2, 3, 4***  In the past 4 months, have you used social media, for example, Facebook, WhatsApp, Instagram, etc.? If yes, which ones?  ***Multiple options allowed*** | 0. Don’t use any social media  1. Facebook  2. Twitter  3. WhatsApp  4. Instagram  5. YouTube  6. JioChat  7. Messenger Chat  8. WeChat  9. Viber  10. Telegram  95. Others (specify)____  -96. Don’t know about social media |
| **F7b** | ***ASK IF F6 1 – 10 and 95***  What all did you do with social media?  ***Multiple options allowed*** | 1. Phone call  2. Messaging and chatting  3. Money transfer  4. Online shopping  5. Listen to music / watch videos  6. Take/upload pictures/ videos.  7. Play games.  95. Others(specify) |
| **F8b** | In the past 4 months, have you used an ATM machine? | 0. No  1. Yes |
| **F9** | Do you own any house or flat either alone or jointly with someone else? | 0. Do not own  1. Yes, own alone  2. Yes, own Jointly  3. Yes, own alone and jointly |
| **F10** | Do you own any agricultural or non-agricultural land either alone or jointly with someone else? | 0. Do not own  1. Yes, own alone  2. Yes, own Jointly  3. Yes, own alone and jointly |

| **READ: Lets talk about household decision making** | | |
| --- | --- | --- |
| **QN** | **Questions** | **Codes** |
| **F11** | Who decides how your husband's earnings will be used?  (***Multiple choice)*** | 1. Respondent (Wife)  2. Husband  3. MIL/FIL  4. other relatives  96. Husband has no earnings ***(CAPI show only this option if B7 = No or B8 = 0)*** |
| **F12** | Who usually makes decisions about making major household purchases?  (***Multiple choice)***  (For e.g.: Furniture, appliances, electronics, vehicles etc.). | 1. Respondent (Wife)  2. Husband  3. MIL/FIL  4. other relatives |
| **F13** | Who decides about visits to your family or relatives?  (***Multiple choice)*** | 1. Respondent (Wife)  2. Husband  3. MIL/FIL  4. other relatives |
| **F14** | Who usually decides how your earnings will be used?  (***Multiple choice)*** | 1. Respondent (Wife)  2. Husband  3. MIL/FIL  4. other relatives  96. respondent has no earnings |
| **F15** | Who usually makes decisions about health care for yourself?  (***Multiple choice)*** | 1. Respondent (Wife)  2. Husband  3. MIL/FIL  4. other relatives |

| **READ: Lets talk about the places you can visit** | | |
| --- | --- | --- |
| **QN** | **Questions** | **Codes** |
| **F16** | Are you usually allowed to go to the following places alone or only with someone else, or not at all? | 0. Not at all  1. Yes, Alone  2. Yes, with someone else |
| a | To the market in the village | 0 1 2 |
| b | Meeting friends/relatives staying in the village | 0 1 2 |
| c | To a nearby health facility (which can be away from the village) | 0 1 2 |
| d | To places outside the village/panchayat (for any reason / purpose) | 0 1 2 |
| e | Mela/Fair nearby the village | 0 1 2 |

|  | **READ: Below is a list of statements dealing with your general feelings about yourself. Please indicate how strongly you agree or disagree with each statement. Please note that some statements may appear repetitive or of the same meaning to you, but it is intentional, and we apologize for making you answer for similar statements.** | |
| --- | --- | --- |
| **F17** | **Codes:**  1. Strongly disagree  2. Disagree  3. Cannot decide /Don’t know  4. Agree  5. Strongly agree | |
| a | On the whole, you are satisfied with yourself | 1 2 3 4 5 |
| b | At times you think you are no good at all. | 1 2 3 4 5 |
| c | You feel that you have a number of good qualities. | 1 2 3 4 5 |
| d | You are able to do things as well as most other people. | 1 2 3 4 5 |
| e | You certainly feel useless at times. | 1 2 3 4 5 |
| f | You feel that you are a person of worth, at least on an equal plane with others. | 1 2 3 4 5 |
| g | You wish you could have more respect for yourself | 1 2 3 4 5 |
| h | All in all, you are inclined to feel that you are a failure. | 1 2 3 4 5 |
| i | You take a positive attitude toward yourself | 1 2 3 4 5 |
| j | You feel you do not have much to be proud of. | 1 2 3 4 5 |
| **F18** |  | |
| A | It is easy for you to stick to your aims and accomplish your goals. | 1 2 3 4 5 |
| b | If someone opposes you, you can find the means and ways to get what you want. | 1 2 3 4 5 |
| c | You can remain calm when facing difficulties because you can rely on your coping abilities. | 1 2 3 4 5 |
| d | You can **always** manage to solve difficult problems if I try hard enough | 1 2 3 4 5 |
| e | If you are in trouble, you can usually think of a solution | 1 2 3 4 5 |
| f | You can solve most problems if you invest the necessary effort. | 1 2 3 4 5 |
| g | When you are confronted with a problem, you can usually find several solutions | 1 2 3 4 5 |
| h | You can usually handle whatever comes your way. | 1 2 3 4 5 |
| i | You are confident that you could deal efficiently with unexpected events. | 1 2 3 4 5 |
| j | Thanks to your resourcefulness, you know how to handle unforeseen situations. | 1 2 3 4 5 |

**Women’s and Girls’ Empowerment in Sexual and Reproductive Health Index**

**READ: Now I’m going to ask you for a series of statements about family planning and contraception. Pease indicate how strongly you agree or disagree with each statement. Please note that some statements may appear repetitive or of the same meaning to you, but it is not intentional, and we apologize for making you answer for similar statements.**

|  |  | **Codes:**  1. Strongly disagree  2. Disagree  3. Cannot decide /Don’t know  4. Agree  5. Strongly agree |
| --- | --- | --- |
| F40 | You can decide when to get pregnant |  |
| F41 | You can decide to have a gap between pregnancies (such as time between your first and second child). |  |
| F42 | If you use contraceptive methods such as goli/ tablet, condom/rubber, injection to plan your family, your husband may seek another sexual partner |  |
| F43 | There could be/will be a conflict with your husband if you use contraceptive methods (goli/ tablet, condom/rubber, injection) for family planning. |  |
| F44 | There could be/will be conflict in your relationship with your mother-in-law if you use contraceptive methods (goli /tablet, condom/rubber, injection) for family planning. |  |
| F45 | If you use contraceptive methods (such as goli /tablet, condom/rubber, injection) your body may experience side effects. |  |
| F46 | Side effects from contraceptive methods (goli/ tablet, condom/rubber, injection) may disrupt your relations with your husband |  |
| F47 | If you refuse sex with your husband, he will be upset with you. |  |
| F48 | If you refuse sex with your husband, he will be hurt or beat you. |  |
| F49 | If you refuse sex with your husband, he will force you to have sex. |  |
| F50 | If you show (express to) your husband that you want to have sex, he will consider you shameless. |  |
| F51 | If you refuse sex with your husband, he will stop giving you money. |  |
| F52 | You have the freedom to choose when you want to begin or stop having children |  |
| F53 | You can decide when to start having children. |  |
| F54 | You can decide how many children to have. |  |
| F55 | Your husband and you can make a joint decision about when to begin having children. |  |
| F56 | You can discuss with your husband about how many children to have. |  |
| F57 | You have a sense of ease that you can discuss with your mother-in-law, when to begin having children. |  |
| F58 | You have a sense of ease that you can discuss using contraceptive methods (such as goli/ tablet, condom/rubber, injection) for family planning with your husband. |  |
| F59 | You can decide when to begin a new contraceptive method (such as such as goli /tablet, condom/rubber, injection) if you want to. |  |
| F60 | You can decide to switch from one contraceptive method to another if you want to (such as such as goli/tablet, condom/rubber, injection). |  |
| F61 | When it comes to choosing a contraceptive method (such as goli/ tablet, condom/rubber, injection) you are confident in telling your healthcare provider (doctor or Asha) what you want. |  |
| F62 | You are confident you can tell your husband when you want to have sex. |  |
| F63 | You have the autonomy (freedom) to decide when to have sex. |  |
| F64 | If you do not want to have sex, you can tell your husband. |  |
| F65 | If you do not want to have sex, you am able to avoid it with your husband. |  |

# SECTION G: RELATIONSHIP QUALITY

**READ: Now I will ask questions about conversations you and your husband have**

| **QN** | **Questions** | **Codes** |
| --- | --- | --- |
| **G1** | Please rate **how happy** you are in your relationship with your husband?  **CAPI- Please use smileys** | 1. Extremely happy  2. Very happy  3. Cannot decide / Don’t know  4. Somewhat happy  5. Not happy at all |
| **G2** | In the past month, how many times have you had a pleasant conversation with your husband? | 1. All the time  2. several times  3. sometimes  4. not at all |
| **G3** | In the past month, how many times has your husband asked you about your likes and dislikes and difficulties this month? | 1. All the time  2. several times  3. sometimes  4. not at all |
| **G4** | In the past month, how often has your husband sought your advice/support in making important decisions? | 1. All the time  2. several times  3. sometimes  4. not at all |
| **G5** | In the past month, how often have you and your husband had dinner together? | 1. All the time  2. several times  3. sometimes  4. not at all |
| **G6** | In the past month, how often have you and your husband gone out together?  *Hint: For example, visited the temple, gone to a movie or a market, visited a friend etc.* | 1. All the time  2. several times  3. sometimes  4. not at all |
| **G7** | In the past month, how often has your husband helped you with household chores such as fetching water, cleaning house, shopping, cooking etc.? | 1. All the time  2. several times  3. sometimes  4. not at all |
| **G8** | In the past month, how often have you argued with your husband? | 1. All the time  2. several times  3. sometimes  4. not at all |
| **G9** | ***Ask if G8 = 1, 2 or 3***  In the past month, what issues did you and your husband have arguments about?  ***Multiple response possible*** | 1. Money / Finance related  2. About having children  3. Other family members related issues (in-laws)  4. Drinking, smoking habits  5. About work type, work hours  6. Household chores  7. Due to commuting  95. Others |
| **G10** | ***Ask if G8 = 1, 2 or 3***  In most of the cases, how was the argument resolved? | 1. We did not resolve it  2. We agreed upon a solution together  3. Husband agreed with me  4. I agreed with him  95. Others |

**READ: Now I will ask same questions as above but about your con versations with your mother-in-law**

**(B). Relationship Quality with Mother-in-Law**

**READ: Now I will ask same questions as above but about your con versations with your** [**MIL Name**]

| **G11** | Would you say that your relationship with your mother-in-law is extremely happy, very happy, neutral, somewhat happy, or not happy at all? | 1. Extremely happy  2. Very happy  3. Cannot decide / Don’t know  4. Somewhat happy  5. Not happy at all |
| --- | --- | --- |
| **G12** | In the past month, how many times have you had a pleasant conversation with your mother-in-law? | 1. All the time  2. several times  3. sometimes  4. not at all |
| **G13** | In the past month, how many times has your mother-in-law asked you about your likes and dislikes and difficulties this month? | 1. All the time  2. several times  3. sometimes  4. not at all |
| **G14** | In the past month, how often has your mother-in-law sought your advice/support in making important decisions? | 1. All the time  2. several times  3. sometimes  4. not at all |
| **G15** | In the past month, how often have you and your mother-in-law had dinner together? | 1. All the time  2. several times  3. sometimes  4. not at all |
| **G16** | In the past month, how often have you and your mother-in-law gone out together?  Hint: For example, visited the temple, gone to a movie or a market, visited a friend etc | 1. All the time  2. several times  3. sometimes  4. not at all |
| **G17** | In the past month, how often has your mother-in-law helped you with household chores such as fetching water, cleaning house, shopping, cooking etc | 1. All the time  2. several times  3. sometimes  4. not at all |
| **G19** | In the past month, how often have you argued with your mother-in-law? | 1. All the time  2. several times  3. sometimes  4. not at all |
| **G20** | ***Ask if G19 = 1, 2 or 3***  In the past month, about what issues did you and your mother-in-law have arguments about?  ***Multiple response possible*** | 1. Money / Finance related  2. About having children  3. Other family members related issues (in-laws)  4. Drinking, smoking habits  5. About work type, work hours  6. Household chores  7. Due to commuting95. Others |
| **G21** | ***Ask if G19 = 1, 2 or 3***  In most of the cases, how was the argument resolved? | 1. We did not resolve it  2. We agreed upon a solution together  3. Mother-in-law agreed with me  4. I agreed with her  95. Others |

**Enumerator: Intimate Partner Violence related questions. Please ensure privacy before asking the following questions**

***READ****:* **Now I would like to ask your personal questions about whether and how your husband physically hurt you. You may find some of these questions discomforting but let me remind you that your answers are completely confidential. You also can refuse answer to any statement, and I will go on to the next statement. If you need any help in this matter, I can also refer you to a local NGO who can help you.**

| **QN** | **Questions** | **Codes** |
| --- | --- | --- |
| G22b | I will read out some statements and you please tell me whether and how often did your husband do the following in the past 4 months | 1. Often  2. Sometimes  3. Rarely  4. Never  -98. Refused answer |
| A | Say or do something to humiliate you in front of others? | 1 2 3 4 -98 |
| B | Threaten to hurt or harm you or someone else close to you? | 1 2 3 4 -98 |
| C | Insult you or make you feel bad about yourself? | 1 2 3 4 -98 |
| D | Push you, shake you, or throw something at you? | 1 2 3 4 -98 |
| E | Has beaten you physically such as Twist your arm/ pull your hair/slap you/punch you/kick you/drag you? | 1 2 3 4 -98 |
| F | Has he used any kind of weapon to attack you? | 1 2 3 4 -98 |
| G | Physically force you to have sexual intercourse/sexual acts with him when you did not want to? | 1 2 3 4 -98 |
| G23b | In the past 4 months, were you afraid of your husband: most of the time, sometimes, or never? | 0. Never  1. Most of the times  2. Sometimes |

# SECTION H: ASPIRATIONS AND HOPE

| **QN** | **Questions** | **Codes** |
| --- | --- | --- |
| **H1** | What are your dreams in life in the next one year about education, if any?  **Multiple response possible** | 1. No goal / plan 2. Continuing my school / college. 3. Complete my education through correspondence (remote) 4. Get vocational / skills training. (eg, sewing, knitting, embroidery etc.)   95. Other |
| **H2** | What are your dreams in the next one year about employment or earning income, if any?  **Multiple response possible** | 0. No dreams  1.Learn employable/income-generating skills like sewing, knitting, embroidery, etc.  2. earn more than now  3. Get a job outside the home  4. Get at-home job (cottage industry)  5. Help in family business / shop / farming  95 Other |
| **H3** | What are your dreams in the next one year about your marriage and childbearing, if any?  **Multiple response possible** | 0. No dreams   1. Strengthen relationship with my husband. 2. Improve relationships with MIL, FIL/other relatives. 3. My husband should stay at home longer / less migration/travel. 4. Get pregnant / Have a baby. 5. Not getting pregnant / Using family planning to delay pregnancy.   95. Other |
| **H4** | Are there any other dreams in the next one year which we have not discussed above? Anything else you want?  **Multiple response possible** | 1. No dreams 2. Save money. 3. Improve my health. 4. Visit my family. 5. Be happy.   95.  Other |
| **H5** | Please indicate to what extent the following statements describe your personality and thoughts. Is it Not at all, a little, somewhat, and a lot | 0. Not at all  1. A little  2. Somewhat  3. A lot  -99 Don’t know / cannot say |
| a | You expect good things to happen to you | 0 1 2 3 -99 |
| b | You are excited about your future | 0 1 2 3 -99 |
| c | You trust your future will turn out well | 0 1 2 3 -99 |

# SECTION I: BELIEFS AND NORMS

**ATTITUDES AND BELIEFS**

**READ: I am now going to read a few statements about your own beliefs or opinions about husbands and wives. Everybody’s opinions are their own so that there are no right or wrong answers. Therefore, truthfully indicate how strongly you agree or disagree with the following statements.**

| **Codes:**  1. Strongly disagree  2. Disagree  3. Cannot decide /Don’t know  4. Agree  5. Strongly agree | | |
| --- | --- | --- |
| **I1** | Family planning is women's business, and a man should not have to worry about it. | 1 2 3 4 5 |
| **I2** | Women who use contraception may become characterless/shameless. | 1 2 3 4 5 |
| **I3** | It is wrong to use family planning methods to avoid or delay pregnancy. | 1 2 3 4 5 |
| **I4** | It is wrong to use family planning methods to avoid or delay pregnancy before birthing the first child. | 1 2 3 4 5 |

**READ: Now I will ask you some extremely sensitive questions, to which you have to answer only “yes” or “no”. Let me assure you again that your answers are completely confidential and will not be told to anyone. If we should come to any question that you don't want to answer, just let me know and we will go to the next question. Please answer as No or yes.**

| **Q** |  | 1. No 2. Yes   -99 Don’t know |
| --- | --- | --- |
| **I5** | If a wife knows her husband has a sexually transmitted disease, is she justified in asking that they use a condom when they have sex? | 0 1 -99 |
| **I6** | If a wife knows her husband has sex with other women, is she justified in refusing to have sex with him? | 0 1 -99 |
| **I7** | Can a wife say ‘no’ to her husband if she does not want to have sexual intercourse with him? | 0 1 -99 |
| **I8** | Do you think that if a wife refuses to have sex with her husband when he wants her to, he has the right to: |  |
| a | Angry and reprimand her? | 0 1 -99 |
| b | Refuse to give her money or other means of financial support? | 0 1 -99 |
| c | Use force to have sex with her even if she doesn’t want to? | 0 1 -99 |
| d | Have sex with another woman? | 0 1 -99 |
| **I9** | In your opinion, is a husband justified in hitting or beating his wife in the following situations: |  |
| a | If she goes out without telling him? | 0 1 -99 |
| b | If she neglects the house or the children? | 0 1 -99 |
| c | If she argues with him? | 0 1 -99 |
| d | If she refuses to have sex with him? | 0 1 -99 |
| e | If she doesn't cook food properly? | 0 1 -99 |
| f | If he suspects her of being unfaithful? | 0 1 -99 |
| g | If she shows disrespect for in-laws? | 0 1 -99 |

**DESCRIPTIVE NORMS**

**READ**: This section asks about what **most families are doing in your opinion**. We understand that you may not be aware of what families you know are doing, but we are asking what you think they are doing. For example, you may believe or know that most of the families you know “actually” use toilet. In this case, you will strongly agree. If you have no belief or knowledge on whether people wash hands with soap with soap, you can say “I don’t know, or I cannot decide”. Remember, there is no right or wrong answer. Now I will read each sentence and indicate how strongly you agree or disagree.

| **Codes:**  1. Strongly disagree  2. Disagree  3. Cannot decide /Don’t know  4. Agree  5. Strongly agree | | |
| --- | --- | --- |
| **I11** | Taking care of children is only women’s job in most of the families you know in this village | 1 2 3 4 5 |
| **I12** | Men are the only ones who earn money in most of the families you know in this village | 1 2 3 4 5 |
| **I13** | Women are beaten in certain circumstances in most of the families you know in this village. | 1 2 3 4 5 |
| **I14** | Women obey their husbands in all matters in most of the families you know in this village | 1 2 3 4 5 |
| **I15** | Women ask permission from their husbands to get medical treatment of any kind in most of the families you know in this village | 1 2 3 4 5 |
| **I16** | Husbands make the decision about buying major household items (e.g., television, refrigerator, bicycle, motor bikes) in most of the families you know in this village | 1 2 3 4 5 |
| **I17** | Women eat last after all the family members have eaten in most of the families you know in this village | 1 2 3 4 5 |
| **I18** | Women eat left over food after the rest of their family has eaten in most of the families you know in this village | 1 2 3 4 5 |

**INJUNCTIVE NORMS**

**READ**: This section asks about your opinion about what are the beliefs of most of the people in your village. Unlike before, we are not asking your opinion on what people are actually doing but we are asking your opinion about what people “believe” in. For example, in your opinion, most people may believe that using toilets is good for their health, but in reality, very few are using toilets. In this case, you should strongly agree with the statement that ‘most people believe that toilet should be used”. Sometimes, it is possible that you don’t have any opinion or idea about whether most people believe in something. In that case you can answer “I don’t know, or I cannot decide”. Remember, there is no right or wrong answer. Now I will read each sentence and indicate how strongly you agree or disagree.

| **Codes:**  1. Strongly disagree  2. Disagree  3. Cannot decide/don’t know  4. Agree  5. Strongly agree | | |
| --- | --- | --- |
| **I19** | Most of the villagers believe that it *should* only be a woman’s job to take care of the children | 1 2 3 4 5 |
| **I20** | Most of the villagers believe that men *should* be the only ones who earn money for the family | 1 2 3 4 5 |
| **I21** | Most of the villagers believe that women should be beaten in certain circumstances. | 1 2 3 4 5 |
| **I22** | Most of the villagers believe that women *should* obey their husbands in all matters. | 1 2 3 4 5 |
| **I23** | Most of the villagers believe that women *should* ask permission from their husbands to get medical treatment of any kind | 1 2 3 4 5 |
| **I24** | Most of the villagers believe that husbands *should* make the decision about buying major household items (e.g., television, refrigerator, bicycle, motor bikes) | 1 2 3 4 5 |
| **I25** | Most of the villagers believe women should eat last, after all the family members have eaten. | 1 2 3 4 5 |
| **I26** | Most of the villagers believe women should eat left over food after the rest of their family has eaten food. | 1 2 3 4 5 |

# SECTION J: ANXIETY AND DEPRESSION

| **J1** | The following questions are related to certain pains and problems. Describe how often you were troubled by the following symptoms **during the last 2 weeks**.  You can answer *not at all, few days (1-6 days), more than a week (7-11 days), nearly every day (12-14 days).* | |
| --- | --- | --- |
|  | **Codes:**  0. Not at all   1. Few days (1-6 days) 2. More than a week (7-11 days) 3. Nearly every day (12-14 days) | |
| a | Little interest or pleasure in doing things | 0 1 2 3 |
| b | Feeling down, depressed, or hopeless | 0 1 2 3 |
| c | Trouble falling or staying asleep, or sleeping too much | 0 1 2 3 |
| d | Feeling tired or having little energy | 0 1 2 3 |
| e | Poor appetite or overeating | 0 1 2 3 |
| f | Feeling bad about yourself — or that you are a failure or have let yourself or your family down | 0 1 2 3 |
| g | Trouble concentrating on things, such as reading the newspaper or watching television | 0 1 2 3 |
| h | Moving or speaking so slowly that other people could have noticed? Or the opposite — being so fidgety or restless that you have been moving around a lot more than usual | 0 1 2 3 |
| i | Thoughts that you would be better off dead or of hurting yourself in some way | 0 1 2 3 |
| **J2** | ***Ask if J1 =1, 2, 3 for any of a-I***  You have answered that some of the above problems have troubled you in the past 2 weeks. How difficult have these problems made it for you to do your usual work, take care of things at home or get along with other people? | 0. Not difficult at al   1. Little difficult 2. More difficult 3. Very difficult |

# SECTION K: KNOWLEDGE

**READ: Now I am going to ask you a few questions about your knowledge regarding family planning, birth / pregnancy.**

| **FAMILY PLANNING** | | | |
| --- | --- | --- | --- |
| QN. | Questions | | Codes |
| **K1** | What is the recommended minimum gap in months between two children by health experts or government advertisements? | | Months: _________ ***(Range: 1 to 72 months)***  -77 more than 72 months (more than 6 years)  -99 Don’t know |
| **K2** | What are the benefits of keeping a gap of at least 3 years between two children?    ***Multiple response possible*** | | 1. Reduced risk of infant and child mortality 2. Improved child development and well-being 3. Enhanced maternal health and well-being. 4. Reduced risk of maternal complications during pregnancy and childbirth 5. Increased likelihood of healthy birth outcomes 6. Improved spacing between pregnancies for maternal physical and mental recovery. 7. Enhanced bonding and nurturing between mother and child. 8. Increased family planning options and choices 9. Improved educational opportunities for both children and parents. 10. Better economic stability and resources for the family 11. Reduced risk of preterm birth and low birth weight 12. Reduced risk of maternal anemia and nutritional deficiencies 13. Facilitation of breastfeeding and optimal infant feeding practices 14. Reduced risk of unintended pregnancies   95.Others (specify)  -99 Don’t know |
| **K3** | What are some of the temporary family planning methods a couple can use to give space between births or **DELAY** pregnancy?  ***Multiple Response possible*** | | 1. Copper T  2. Antara Injections (Such as Depo Provera)  3. Emergency pills (taken within 3 days)  4. Everyday pills (such as Mala-N, Chhaya)  5. Male condom  6. Female condom  7. Safe/Standard days method/ Rhythm method  8. Withdrawal  95. Other method, specify---------  -99. Don't know |
| **K4** | What are some of the family planning methods a couples can use to **AVOID** children permanently?  ***Multiple response possible*** | | 1. Copper T  2. Antara (such as Depo Provera)  3. Emergency pills (taken within 3 days)  4. Everyday pills (such as Mala-N, Chhaya)  5. Male condom  6. Female condom  7. Safe/Standard days method/ Rhythm method  8. Withdrawal  9. Female sterilization  10. Male sterilization  11. Hysterectomy  95. Other method, specify---------  -99. Don't know |
| **K5** | From one menstrual period to the next, if a woman has sexual relations, when is a woman more likely to become pregnant?  *Hint: Read out the options for the respondents.* | | 1. Just before her period begins  2. During her period  3. Right after her period has ended  4. Halfway between two periods  5. All days are equal  -96. Menstruation and pregnancy are not related to each other.  -99 Don’t know |
| **K6** | Let's assume a woman has recently given birth to a child and the child is 4-months old and her periods (menstrual cycles) haven't come back, and she is exclusively breastfeeding her 4-month child both during the day and night. Do you know if such a woman gets pregnant if she has unprotected sex? | | 0. No  1. Yes  -99. Don’t know. |
| **K7** | Before becoming pregnant, what actions must a woman take to improve her health in preparation for the pregnancy?  ***Multiple responsible possible*** | 0. Do nothing.  1. Take folic acid (Red tablets)  2. Stop or cut down smoking.  3. Stop or cut down drinking alcohol  4. Eat more healthily  5. Seek medical/health advice.  6. Took pre-natal vitamins  7. Decrease physical activity / take rest  8. Increase physical activity / work  9. Reduce Tea/Coffee intake  10. Visit a healthcare provider for preconception counseling / check up  11. Start a regular exercise routine  12. Stop or reduce use of certain medications  13. Be happy / stress free  95. Other (Specify)  -99 Don’t know | |
| **K8** | In your opinion, what should a pregnant woman do to take care of her and her baby’s health?  ***Multiple response possible*** | 1. Consume a variety of foods including green vegetables, pulses, etc. 2. Reach and maintain a healthy weight. 3. Take adequate rest during the day and night. 4. Take iron and folic acid supplements. (Red tablets) 5. Take calcium supplements. (White tablets) 6. Avoid heavy/strenuous work. 7. Attend regular prenatal check-ups with a healthcare provider. 8. Get tetanus toxoid immunization. 9. Stay hydrated by drinking an adequate amount of water. 10. Avoid smoking, alcohol, and illicit drugs. 11. Seek emotional support and reduce stress levels. 12. Follow any prescribed medications or treatments. 13. Attend childbirth education classes to prepare for delivery. 14. Seek guidance on breastfeeding and newborn care. 15. Discuss any concerns or questions with a healthcare provider. 16. Not have sexual relations with husband   -99. Don’t Know  95. Others (specify | |
| **K9** | Do you think consumption of iron folic acid supplementation (or eating IFA tablets) is important during pregnancy?  (Red tablets) | 0. No  1.Yes ***(CAPI - Only show this option with default selection if K K8 = 4)***  -96 Don’t know if IFA is important or not.  -99 don’t know what IFA is. | |
| **K10** | Is abortion legal in India? | | 0. No  1. Yes  2. Yes, it depends on the month of pregnancy  -99. Don’t know/ can't say for sure |
| **K11** | Is sex determination of the child before childbirth legal in India? | | 0. No  1. Yes  -99. Don’t know/ can't say for sure |
| **K12** | How do you think the sex of a child (boy or girl) is determined?  ***Multiple response possible*** | | 1. God's will 2. Time of month when conceived. 3. Position used before pregnancy. 4. What does the stomach look like during pregnancy (raised up or sitting down) 5. Religious factors - Puja, Mantra, Prasad, Blessings 6. History of boys and girls in the woman's family 7. Women's Health or Genetics 8. History of boys and girls in a man's family 9. Men’s health or genetics 10. If you have a child in 9 months, then a girl, otherwise a boy. 11. It is determined by chance/not in anyone’s control.   95. other (specify______)  -99. Don’t know |

# SECTION L: TIME USE

***Study time allocation***

| ***Q No*** | ***Question*** | ***Codes*** |
| --- | --- | --- |
| **L1** | Interviewer: Confirm whether the respondent is currently enrolled in any school, college, or any other education facilities. | [ 0] No ***(Skip to L5)***  [ 1] Yes |
| **L2** | Thinking of the last 30 days, how many days would you say you have studied or done anything related to your course work? | [ ][ ] days ***Range 0 to 30 days***  (-99) Don’t know. |
| **L3** | ***Ask if L2> 0.***  Thinking of the last 30 days, how many hours in a typical day (in 24 hrs.) do **you** study or do anything related to your course work? | [ ][ ] hours/day ***Range 0 to 15 hours***  (-99) Don’t know. |
| **L4** | ***Ask if E4 = 1***  Compared to the situation before getting pregnant, are you studying for more, less or the same hours? And why?  ***(Single Code)*** | (0) No difference in study hours  (1) Increased study hours due to pregnancy/baby  (2) Decreased study hours due to pregnancy/baby  (3) Increased study hours due to other reasons  (4) Decreased study hours due to other reasons  (-9) Don’t know |

***ii. Earning/Job Work Time Allocation***

| ***Q No*** | ***Question*** | ***Codes*** |
| --- | --- | --- |
| **L5** | Interviewer: Please confirm if the respondent currently working to earn income or looking for a job? | 0. Does not work and does not even look for work *(Skip to L9)*  1. Not working but looking for work  2. works |
| **L6** | ***Ask if L5=1 or 2***  Thinking of the last 30 days, how many days would you say you have worked to earn any income or were looking for any job to earn some income? | [ ][ ] days **Range 0 to 30 days**  (-99) Don’t know. |
| **L7** | ***Ask if L6> 0***  Thinking of the last 30 days, how many hours in a typical day (in 24 hours) did **you** work to earn any income or spend time to search for any job to earn some income? | [ ][ ] hours **Range 0 to 15**  (-99) Don’t know. |
| **L8** | ***Ask if E4 = 1***  Compared to the situation before getting pregnant, are you spending more, less or some hours in work to earn income or searching for a job? And why?  ***(Single Code)*** | 0. No difference in earning income or time spent searching for a job.  1. Increase in time spent earning income or looking for a job due to pregnancy/child.  2. Reduced time spent earning income or looking for a job due to pregnancy/child.  3. Increase in time spent earning income or looking for a job for other reasons.  4. Reduction in time spent earning income or searching for a job due to other reasons.  -99 Don’t know |

**iii. Own Housework Time Allocation**

| **Q No** | ***Question*** | ***Codes*** |
| --- | --- | --- |
| **L9** | Thinking of the last 30 days, how many days would you say you have done household chores such as taking care of people, cooking, cleaning? | [ ][ ] days **Range 0 to 30 days**  (-99) Don’t know. |
| **L10** | ***Ask if L9> 0***  Thinking of the last 30 days, how many hours in a typical day (in 24 hrs) did **you** spend in household chores? | [ ][ ] hours/day **Range 0 to 15 hours**  (-99) Don’t know. |
| **L11** | ***Ask if E4 = 1***  Compared to the situation before getting pregnant, are you spending more, less or the same hours in household chores? And why? ***(Single Code)*** | 0. No difference in the time spent on household chores.  1. Increase in time spent on household chores due to pregnancy/child.  2. Reduced time spent on household chores due to pregnancy/child.  3. Increase in time spent on household chores due to other reasons.  4. Reduction in time spent on household chores due to other reasons.  (-99) Don't know. |

**iv. Leisure time (meeting friends, watching TV)**

| ***Q No*** | ***Question*** | ***Codes*** |
| --- | --- | --- |
| **L12** | Thinking of the last 30 days, on many days have you done some activities to entertain and relax yourself such as meeting friends, going for a movie, watching TV, social media, or videos on phone, going to some social function etc.? But these activities do not include afternoon nap or night sleep | [ ][ ] days **Range 0 to 30 days**  (-99) Don’t know. |
| **L13** | ***Ask if L12> 0***  Thinking of the last 30 days, how many hours in a typical day (in 24 hrs.) do **you** spend on activities that entertain or relax you? (Excluding naps and sleep) | [ ][ ] hours/day **Range 0 to 15 hours**  (-99) Don’t know. |
| **L14** | ***Ask if E4 = 1***  Compared to the situation before getting pregnant, are you spending more, less or the same hours in household chores? And why? ***(Single Code)*** | 0) No difference in hours to entertain yourself or rest  (1) Increased hours of self-entertainment or rest due to pregnancy/child  (2) Decreased hours of leisure or entertainment due to pregnancy/child  (3) Increase in hours of rest or entertainment for other reasons  (4) Decreased hours of leisure or entertainment due to other reasons  (-9) Don’t know |

# Section M: TARANG INTERVENTION MEASURES

You recently participated in our reproductive health empowerment intervention called TARANG, we would like to know more about your experience with it.

|  |  | | |  |
| --- | --- | --- | --- | --- |
| **Q. N** | **Questions** | **Coding Categories** |  | **Skip** |
|  | In each question, I will ask you to provide a score of 1-5 and explain what scores you would give for each of the following questions. | | |  |
| M0 | How many TARANG group training and discussion sessions have you attended since it was launched in August 2023?  (Enumerator: help respondent recall by asking about the most recent session, then how many in the current month, the month before, and so on. Estimate the total number and get confirmation from the respondent) | Number: (Range 0-14)  CAPI: Range as per the session plan already shared w you. | |  |
| M1 | Please tell us on a scale of 1 to 5, how satisfied were you overall with the Tarang program? where 1 means "not satisfied at all" and 5 means "completely satisfied”.? | 1. Completely dissatisfied / Completely unsatisfied  2. Somewhat dissatisfied  3. Neither satisfied nor dissatisfied  4. Somewhat satisfied  5. Completely satisfied |  |  |
| M2 | On a scale of 1 to 3, please tell us how useful you found the discussions and topics discussed during the Tarang sessions, where 1 means "not useful at all," 2 means "somewhat useful," 3 means "very useful," or you can choose not to say. | 1. Not useful at all  2. Somewhat useful  3. Very useful  4. Can't say anything. |  |  |
| M3 | On a scale of 1 to 3, please tell us how knowledgeable you found the person providing training (trainer) during the Tarang program, where 1 means "not knowledgeable at all," 2 means "somewhat knowledgeable," 3 means "very knowledgeable," or you can choose not to say | 1. Not knowledgeable at all  2. Somewhat knowledgeable  3. Very knowledgeable  4. Can't say anything |  |  |
| M4 | On a scale of 1 to 3, please tell us how likely it is that you will recommend a friend to join the Tarang program, where 1 means "very unlikely," 2 means "somewhat likely," 3 means "very likely," or you can choose not to say | 1. Very unlikely  2. Somewhat likely  3. Very likely  4. Can't say anything |  |  |
| M5 | On a scale of 1 to 3, kindly share your level of connection with the other participants during the Tarang sessions, where 1 means “very little connection," 2 means “some connection," 3 means "very high connection," or you can choose not to say. | 1. Very little connection  2. Some connection  3. Very high connection  4. Can't say anything |  |  |
| M6 | Please tell us about how you felt regarding the length of each session. The session time limit was very short, the session time limit was very long, the session time limit was just right, or you cannot say anything about the time limit. | 1. The session time limit was very short.  2. The session time limit was very long.  3. The session time limit was just right.  4. Can't say anything. |  |  |
| M7 | Please tell us about the interval (time) between the sessions held in the Tarang program. Did you feel that the interval between two sessions was quite short (meaning more sessions were organized in fewer days), or was the interval between two sessions just right, or was the interval between two sessions quite long (meaning session were organized with a significant gap of days) or you cannot say anything about the interval? | 1. The interval between two sessions in the Tarang program was quite short.  2. The interval between two sessions in the Tarang program was just right.  3. The interval between two sessions in the Tarang program was quite long.  4. Can't say anything. |  |  |
| M8 | Please tell us about your perception of the total number of sessions held in the Tarang program so far. Have there been too few sessions, too many sessions, the right number of sessions, or you cannot say anything? | 1. There have been fewer sessions.  2. There have been more sessions.  3. The right number of sessions have taken place.  4. Can't say anything. |  |  |
| M9 | On a scale of 1 to 3, please let us know to what extent you can apply the information acquired from the sessions in the Tarang program to your life. Where 1 means 'cannot apply at all,' 2 means 'can apply to some extent,' 3 means 'can apply to a great extent,' or you cannot say anything. | 1. Cannot apply at all.  2. Can apply to some extent.  3. Can apply to a great extent.  4. Can't say anything. |  |  |
| M10 | Please tell us how you feel about yourself in relation to making decisions about having children and your health after participating in the Tarang sessions. Do you feel less confident, more confident, or the same as before?" | 1. Less confident than before  2. More confident than before  3.. Just as confident as before  4. Can’t say anything |  |  |
| M11 | Have you observed any positive or negative changes in yourself regarding making decisions about having children and family planning after participating in the Tarang program meetings/sessions, or has there been no change? | 1. No positive changes  2. Some positive changes  3. More positive changes  4. Can’t say anything |  |  |
| M12 | Have you observed any positive or negative changes in your husband’s perception (thinking) regarding having children and family planning after his participation in the Tarang sessions, or has there been no change? | 1. No positive changes  2. Some positive changes  3. More positive changes  4. Can’t say anything |  |  |
| M13 | Please rate, on a scale of 1 to 5, the degree to which your involvement in the Tarang sessions has positively impacted communication and understanding between you and your husband regarding family planning. Where 1 means "quite negative impact," 2 means "a little negative impact," 3 means "no impact at all," 4 means "a little positive impact," and 5 means "a very positive impact. | 1. Quite negative impact  2. A little negative impact,  3. No impact at all  4. A little positive impact  5. A very positive impact. |  |  |
| M14 | Now, I will read you some sentences, and you need to tell me to what extent you agree or disagree with these sentences. | | |  |
|  | **Codes:**  1. Completely disagree  2. Disagree  3. Cannot decide/don’t know  4. Agree  5. Completely agree | | |  |
|  |  |  |  |  |
| A. | Did you like the sessions of the Tarang program – do you agree or disagree? To what extent? | 1 2 3 4 5 |  |  |
| . B | Do you want the sessions of the Tarang program to be held in your village - do you agree or disagree? To what extent?. | 1 2 3 4 5 |  |  |

***This is the end of our interview. Thank you very much for your time and sharing your experiences with us. This is very important to us.***
