# Supplementary material for: Feasibility and acceptability of a life skills and reproductive health empowerment intervention for young newly married women in Rajasthan, India: a pre-post convergent mixed methods pilot study
Source: Pilot Feasibility Stud. 2025 Nov 15;11:142. doi: 10.1186/s40814-025-01720-7 (PMC12619424; doi:10.1186/s40814-025-01720-7)
Supplement: Supplementary file 3 — Additional file 3: Interview Guides for Newly Married Women and NGO staff/moderators. [file 40814_2025_1720_MOESM3_ESM.docx]

**Interview Guides for Newly Married Women and NGO staff/moderators.**

### Interview Guide for the Pilot Evaluation Qualitative Study – Participants

**Icebreakers**

1. Can you tell me a little bit about your everyday life. or can you describe a typical day in your life? What are your daily routines, activities, and responsibilities?
2. Can you tell me a little bit about your family – Who are the members of your household? and what are their roles and relationships within the family? (can include the second part or even skip)

As you know, our TARANG program for young married couples and mothers-in-law in this village. I want to learn about your experience of attending this program and get your feedback on it and how you think it has helped you.

1. Can you tell us about involvement with TARANG program?
2. What are your thoughts on the program (probe for how MIL support or engage with the program in case of husband or newly married woman)?
3. What are some things that you like/liked about it?
4. What are some things that you did not /don’t like?
5. How does it feel to attend the sessions?
   1. Add probes: Do specific emotions come to mind, such as happiness or nervousness?
6. What sessions have really been most meaningful for you, and what makes them stand out?
7. How would you like to see the community and other family members involved in this program? (Probe for how in-laws should or should not be included, how peer support could be useful or how other community members could be engaged? Are there particular roles or responsibilities you believe other community members could take on to support this initiative?)
8. Did you face any barriers or facilitators for you attending the sessions?
9. What made it possible for you attend? (This is to get at facilitators and barriers to attendance)
   - 1. E.g., support of in-law (or lack thereof), support of husband (or lack thereof),
     2. E.g., holidays/ free time/leisure/ extra time
10. What has made it easier for you to attend?
11. What made it harder for you to attend? OR what can be done to make it easier for you to attend?
    1. Were you able to overcome those barriers?. If you managed to overcome any of these barriers, could you share how you did so and whether these strategies could be helpful for others?
    2. How could we make it easier for people to attend?
12. Specifically thinking about the following things, what can be done to make the program more relevant or improve the program?
13. Length of the sessions
14. Timing of the sessions
15. Content of the sessions
16. Composition of the group
17. Follow-up and continuity
18. Local context and cultural sensitivity
19. Feedback mechanisms
20. Moderators’ delivery
21. Moderators’ approach to the sessions?
22. What was missing in our sessions that you think we should have covered?
23. What kind of benefits have you seen because of this program?
24. What kind of personal benefits did you have from attending this TARANG program? (probe one by one).
    - 1. Knowledge of family planning / fertility awareness.
      2. Attitudes towards family planning / fertility awareness.
      3. Practices of family planning.
      4. Time to first birth.
      5. Spacing between children.
      6. Changed Attitudes
      7. Ability to negotiate.
      8. Ability to decide for yourself.
      9. Enhanced Practices
25. Let’s talk about benefits as a couple (ONLY for newly married women / husband)
    - 1. Choice of pregnancy
      2. Intention of pregnancy (Aligned Intentions)
      3. Couples’ communication
      4. Marital relationship (Strengthened Relationship)
      5. Shared Decision-making
      6. Social support from husband
26. Let’s talk about benefits as a family.
    - 1. Relationship
      2. Communication
      3. Trust
      4. Support to attend sessions / other events/ freedom of movement.
      5. Decision-making
27. What, if any, did you observe as costs/downsides (expected and unexpected) to attending these sessions? What is it?
    1. Personal costs (such as time/not able to do housework/ attend office)
    2. Relational costs (such as conflict increase, less support of in-laws/husband/family)
28. Thinking of people who are resistant to family planning in your family/household, how can their attitudes be changed to be more receptive to family planning and family planning methods? Have you tried engaging in open and respectful discussions with these family members about family planning? If so, what approaches have you found effective in fostering understanding and acceptance?
    1. Probe: For example, who in your family is resistant to you adopting family planning methods and then think how can their attitudes be changed?
29. Are there any other ideas or comments you have for the TARANG program?
    1. Any topics you would like more information about? Less information about?
    2. If you could suggest any improvement or change to the TARANG program, what would it be, and how do you think it would enhance the program's effectiveness or relevance to the community?
30. To what extent will you recommend TARANG to others in your community?
31. Could you share specific ways in which the TARANG program has directly impacted your life and your family's well-being?

11. Can you describe any strong networks or support systems that have developed among you and your peers who are attending TARANG sessions? How have these relationships benefited you in the context of family planning?

### Interview Guide for the Pilot Evaluation Qualitative Study – Vikalp Moderators

**IDI guide for Vikalp Moderators (Male and Female moderators)**

**Background/experience/motivation of moderators**

1. What is your experience in leading life skills based SRH programs in the past?
2. What motivates you to be a moderator?
3. What specific skills or qualities do you believe are crucial for a moderator in a program like TARANG?

**Qualitative impact/Benefits to participants (explore by each category of participant)**

1. What is your view on the acceptance of TARANG in the villages you lead this program?
   1. Probe: Acceptable?
   2. Appropriate?
   3. Resonated with participants?
   4. Fit their needs?
2. What have you seen as benefits in participants’ lives after participating in such a program?
   1. Probe: Newly married women (How has participation in TARANG empowered newly married women in their daily lives?)
   2. Mothers-in-law (In what ways have mothers-in-law shown support for their daughters-in-law and the program?)
   3. Husbands? (In what ways have husbands become more engaged in promoting family planning and gender equality?)
3. What challenges did you face in delivering these sessions to the following categories of participants? Discuss for each category of participant (MW, MIL, Husbands) (For e.g., Cultural beliefs and norms, engagement and participation, generational differences, traditional beliefs, perceptions of masculinity, decision-making dynamics etc.)
   1. Newly married women
   2. MIL
   3. Husbands

**What in your view are/were challenges for participants? Discuss for each category of participant (MW, MIL, Husbands)**

1. What are the challenges/barriers for participants to be participating in TARANG?
   1. Time constraints
   2. Childcare
   3. Household chores
   4. Other economic activities
   5. Migration
   6. Elderly care
   7. Stigma and Social Pressure
   8. Access to Resources
   9. Health Issues (especially for MILs)
   10. Others

**What in your view are/were enablers for participants?**

1. What enables participants (women/MIL/husbands) to attend such programs?
   1. What are the facilitators for these participants? (For e.g.: Supportive family, program flexibility, etc.)
2. What, if any, did you observe as personal costs/downsides for participants to attend? (For e.g., time constraint, conflicts in family, impact on work, privacy concerns, social pressures etc.)

**Improvements to the program in the future**

1. What are the things that would make a program like this better?
   1. Length of the sessions
   2. Weekly/ bi-weekly sessions?
   3. Timing of the sessions (Time of the day)
   4. Content of the sessions as in more relevant resources
   5. Composition of the group (homogenous v/s mixed groups?)
   6. Age group of participants
   7. Interactive Elements
   8. Local Cultural Sensitivity

**Thank you for sharing these insights with us. What worked and what did not work?**

1. What has worked well in this program?
2. What has not worked well?
3. What part of the content was easy to deliver for you? Why?
4. What part of the content was difficult to deliver for you? Why?
   1. Probe: How can sensitive topics be delivered easily?
5. In your opinion, can you tell me which particular module helped participants the most (discuss this by category of participant)?
   1. Young newly married women in your community feel empowered?
   2. Mothers-in-law feel empowered / supportive of DILs?
   3. Husbands better partners to their wives / empower their wives?
6. What is missing in the content / can be added / changed/ edited, etc.?
7. What kind of activities resonates with participants?
   1. Probe if required: Videos, Audios, Games, Activities – songs, dance, etc.
8. What kind of activities were hardest for participants?
9. What kind of activities were hardest for you to deploy?
10. What should be done to improve success and attendance of participants?
11. How has the community responded to the program, and what is the overall perception of the program's impact on family planning awareness and practices?
12. What steps can be taken to ensure that the program becomes more community-owned, with participants taking on leadership roles within it?

### Interview Guide for the Pilot Evaluation Qualitative Study – NGO staff

**IDI guide for Vikalp Staff Managing and Providing Oversight to TARANG.**

**Background/experience/motivation**

1. What motivates you to be implementing such a program in your communities?
2. What is your vision for this TARANG program?

**Qualitative impact/Benefits to participants (explore by each category of participant)**

1. What is your view on the acceptance of TARANG in the villages you lead this program?
   1. Probe: Acceptable?
   2. Appropriate?
   3. Resonated with participants?
   4. Fit their needs?
2. What have you seen as benefits in participants’ lives after participating in such a program?
   1. Probe: Newly married women (How has participation in TARANG empowered newly married women in their daily lives?)
   2. Mothers-in-law (In what ways have mothers-in-law shown support for their daughters-in-law and the program?)
   3. Husbands? (Have husbands become more engaged in promoting family planning and gender equality?)
3. What challenges did you observe on the field as you saw moderators delivering these sessions to the following categories of participants? Discuss for each category of participant (MW, MIL, Husbands) (For e.g., Cultural beliefs and norms, engagement and participation, generational differences, traditional beliefs, perceptions of masculinity, decision-making dynamics etc.)
   1. Newly married women
   2. MIL
   3. Husbands

**Based on your observation, what in your view are/were challenges for participants? Discuss for each category of participant (MW, MIL, Husbands)**

1. What are the challenges/barriers for participants to be participating in TARANG?
   1. Time constraints
   2. Childcare
   3. Household chores
   4. Other economic activities
   5. Migration
   6. Elderly care
   7. Stigma and Social Pressure
   8. Access to Resources
   9. Health Issues (especially for MILs)
   10. Others

**Based on your observation, what in your view are/were enablers for participants?**

1. What enables participants (women/MIL/husbands) to attend such programs?
   1. What are the facilitators for these participants? (For e.g.: Supportive family, program flexibility, etc.)
2. What, if any, did you observe as personal costs/downsides for participants to attend? (For eg, time constraint, conflicts in family, impact on work, privacy concerns, social pressures etc)

**Improvements to the program in the future**

1. What are the things that would make a program like this better?
   1. Length of the sessions
   2. Weekly/ bi-weekly sessions?
   3. Timing of the sessions (Time of the day)
   4. Content of the sessions as in more relevant resources
   5. Composition of the group (homogenous v/s mixed groups?)
   6. Age group of participants
   7. Interactive Elements
   8. Local Cultural Sensitivity

**Thank you for sharing these insights with us. What worked and what did not work?**

1. What has worked well in this program?
2. What has not worked well?
3. What part of the content was easy to deliver for moderators? Why?
4. What part of the content was difficult to deliver for moderators? Why?
   1. Probe: How can sensitive topics be delivered easily?
5. What is missing in the content / can be added / changed/ edited, etc.?
6. What kind of activities resonates with participants?
   1. Probe if required: Videos, Audios, Games, Activities – songs, dance, etc.
7. What kind of activities were hardest for participants?
8. What kind of activities were hardest for you to deploy?
9. What should be done to improve success and attendance of participants?
10. What should be done to improve delivery of sessions by moderators?
11. How has the community responded to the program, and what is the overall perception of the program's impact on family planning awareness and practices?
12. What steps can be taken to ensure that the program becomes more community-owned, with participants taking on leadership roles within it?

**High-level questions about TARANG implementation**

1. What kind of challenges did you face with hiring, retaining moderators?
2. If you could start implementing TARANG program again, what would you do differently?
3. What kind of moderators would you hire for the future study?
4. What kind of challenges did you face with recruiting families?
5. How do you envision ensuring the sustainability and growth of the TARANG program in the long term?
6. Anything you want to share with me?
